# Antibacterial Hydrogel Dressing With Ca^2+^‐Dependent Hyaluronidase Responsiveness for Accelerating Wound Healing via On‐Demand Release of AIE Photosensitizers

**DOI:** 10.1002/EXP.20250037

**Published:** 2026-02-26

**Authors:** Rongwei Cui, Hao Yang, Miaomiao Kang, Zhijun Zhang, Weilin Xu, Yang Jiang, Lizhou Xu, Xiao‐Yong Zhan, Luhan Zhang, Yunsheng Xu, Dong Wang, Ben Zhong Tang, Danyang Li

**Affiliations:** ^1^ Research Center, The Seventh Affiliated Hospital Sun Yat‐sen University Shenzhen China; ^2^ ZJU‐Hangzhou Global Scientific and Technological Innovation Center Zhejiang University Hangzhou China; ^3^ Center For AIE Research, Guangdong Provincial Key Laboratory of New Energy Materials Service Safety, College of Materials Science and Engineering Shenzhen University Shenzhen China; ^4^ School of Science and Engineering, Shenzhen Institute of Aggregate Science and Technology The Chinese University of Hong Kong, Shenzhen (CUHK‐Shenzhen) Shenzhen China

**Keywords:** AIE photosensitizer, antibacterial hydrogel, enzyme responsiveness, on‐demand release, wound healing

## Abstract

Reactive oxygen species (ROS)‐assisted photodynamic antibiosis emerges as an effective non‐antibiotic way to eradicate bacteria in infected wounds. To minimize the indiscriminate damage of excessive ROS to normal tissues, on‐demand release of photosensitizers (PSs) is highly desired. Herein, we report a novel aggregation‐induced emission (AIE) PSs‐loaded hydrogel wound dressing by incorporating AIE PSs into hydrogels prepared from sodium alginate and hyaluronic acid (HA) upon crosslinking in Ca^2+^ solution. By virtue of the hyaluronidase (HAase) that is secreted from bacteria, AIE PSs can be released from hydrogels due to the HA degradation. Particularly, the HAase activity can be elevated in the presence of low concentrations of Ca^2+^, where HAase tends to adopt a more stable conformation, whereas high concentrations of Ca^2+^ triggers the salt precipitation of HAase, thereby inhibiting its activity. Benefitting from this advantageous Ca^2+^‐dependent HAase responsiveness, on‐demand release of AIE PSs regulated by the Ca^2+^ that freely diffused from hydrogels is actualized, as evidenced by the accelerated healing of in vivo bacteria‐infected wounds. Overall, this work not only reports the bidirectional regulation mode of Ca^2+^ on HAase activity and elucidates the mechanism for the first time, but also provides a new strategy with on‐demand release of antibacterial PSs for accelerated wound healing.

## Graphical abstract

This study innovates a Ca^2^^+^‐responsive hydrogel dressing that releases AIE photosensitizers on‐demand. Low Ca^2^^+^ enhances bacterial hyaluronidase (HAase) activity, degrading hyaluronic acid dressing to trigger antibacterial ROS release, while high Ca^2^^+^ inhibits HAase, preventing overdosing. This bidirectional Ca^2^^+^ regulation enables on‐demand therapy, minimizing tissue damage and accelerating wound healing.

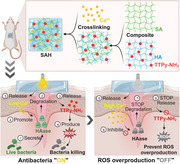

## 1 Introduction

The disease burden of wound care is extensive throughout the world, in which bacterial infections are significant problems in many clinical settings [1, 2, 3]. Due to the overuse of antibiotics, antibiotic‐resistant infections are emerging and posing great hindrance to wound care [4, 5]. Several non‐antibiotic strategies have been developed [6, 7], for example, the traditional Ag^+^ or medical‐grade honey‐based topical therapy [8], the novel photosensitizer‐based photodynamic therapy, the phage‐based antibacterial therapy, electrochemical stimulation‐based biophysical therapy, and commensal bacteria‐based microbial modulation therapy, etc. [9]. Among them, reactive oxygen species (ROS)‐assisted photodynamic inactivation of bacteria is flourishing in recent years with the advantages of noninvasiveness and drug resistance‐free [6, 10, 11]. Despite being effective, undesired damage to normal tissues is usually inevitable, because the excessive photosensitizers (PSs) will generate immoderate ROS in the normal tissues upon light irradiation [12]. In this regard, how to readily prepare high‐performance PSs for efficient photodynamic antibiosis but simultaneously avoid their superfluous release in the wound area is of great concern when managing the infected wounds.

Recent studies have demonstrated that PSs with a unique aggregation‐induced emission (AIE) characteristic (defined as AIE PSs) represent promising candidates for efficient photodynamic antibiosis, as they exhibit a unique aggregation‐enhanced theranostics (AET) property upon aggregation [13]. Beyond the well‐acknowledged aggregation‐enhanced fluorescence, the ROS generation can also be effectively facilitated due to the aggregation‐induced intersystem crossing (AI‐ISC) [14] and more ISC‐directed excited energy decay resulting from the restriction of intramolecular motions in the aggregate state [15, 16]. Besides, the reduced intermolecular π‐π interaction compared to the conventional PSs can further contribute to the fluorescence and ROS channels by decreasing the nonradiative energy dissipation [17]. Up to the present, a good deal of high‐performance AIE PSs have been reported [18] and proved to be capable of killing various pathogens and even multi‐drug resistant strains [19, 20].

In regard to the on‐demand release of PSs, hydrogels have been recognized as an ideal platform. Benefitting from their fascinating features of excellent tissue adhesion, water absorption, responsiveness, and other properties (e.g., anti‐inflammatory and immunological regulation) [21], well‐designed hydrogels have been widely used as wound dressings to promote healing because they can profitably accelerate wound re‐epithelialization and skin regeneration [22]. In particular, responsive hydrogels dressings used as drug carriers can selectively release their payloads in response to trigger mechanisms, thereby enabling a more precise control in the dosage of therapeutic agents and their temporal window for action [23].

On the basis of the individually attractive advantages, combining AIE PSs with hydrogels will be a strong‐strong union strategy to bring new paradigms for infected wound healing [24]. In this respect, for the purpose of efficient photodynamic antibiosis, the AIE PSs should be firstly released from hydrogels to ensure sufficient contact with bacteria due to the short lifespan and limited diffusion distance of ROS [25, 26]. Furthermore, the excessive release of AIE PSs should also be intercepted to avoid the unnecessary toxicity and damage to the normal tissues. However, most of the currently reported studies involving AIE PSs‐loaded hydrogel dressings mainly rely on the uncontrollable release of the PSs upon natural degradation of the hydrogel dressings [27], which are undoubtedly difficult to adapt to the dynamically varying microenvironment of the wounds [28]. Hence, hydrogel dressings that allow on‐demand release of AIE PSs would ensure complete bacteria killing whilst minimizing the side effects caused by ROS overproduction [15, 29].

**SCHEME 1:**
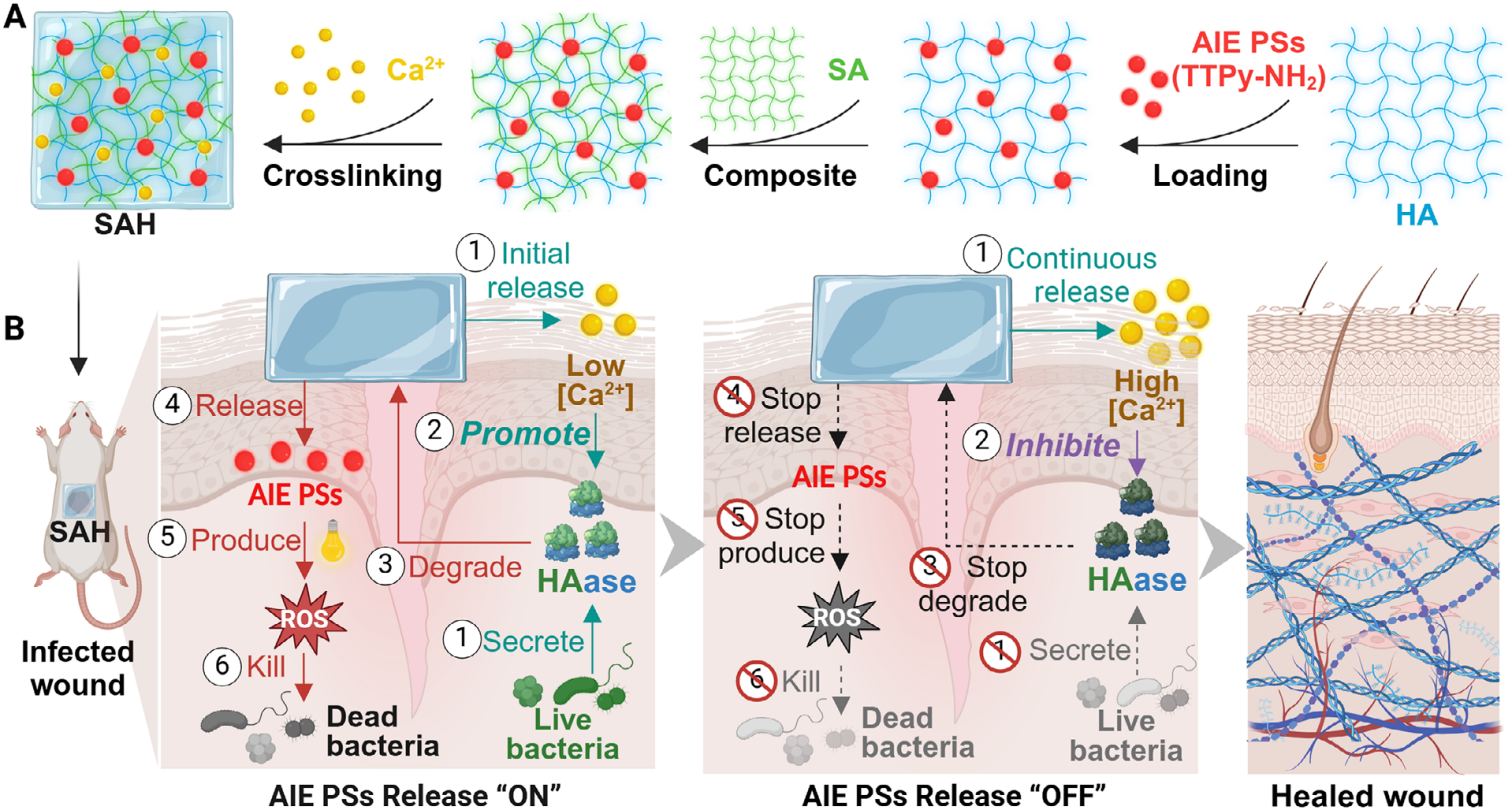
Schematic illustration of a novel AIE PSs‐loaded switchable antibacterial hydrogel (SAH) with Ca^2+^‐dependent HAase responsiveness for on‐demand release of PSs and smart management of infected wounds. (**A**) The fabrication process of SAH involving TTPy‐NH_2_ encapsulation in the HA polymer chain and subsequent Ca^2+^‐mediated gelation. (**B**) The healing of infected wounds via on‐demand and visible light‐triggered production of ROS with a mechanism of Ca^2+^‐dependent HAase responsiveness of SAH dressing.

Herein, we report a novel AIE PSs‐loaded antibacterial hydrogel dressing. As shown in Scheme 1A, the hydrogel dressing was constructed through a sequential fabrication process: (1) loading of AIE PSs (triphenylamine‐thiophene‐pyridinium derivative, TTPy‐NH_2_) to hyaluronic acid (HA), followed by (2) crosslinking of the HA‐composited sodium alginate (SA) matrix with Ca^2^^+^ to form the final switchable antibacterial hydrogel (SAH). Notably, we uncovered a previously unreported regulatory mechanism of Ca^2^^+^, extend beyond their traditional crosslinking role in alginate system. The Ca^2^^+^ exhibit concentration‐dependent modulation of hyaluronidase (HAase, produced by extensive pathogenic bacteria [30]) activity through a feedback loop, enabling precise control over both AIE PSs release and ROS generation, effectively turning these processes on and off as needed.

A typical setting in Scheme 1B demonstrates three distinct phases of antibacterial action of SAH. (1) Pathogen detection phase: infectious bacteria in the wound bed secrete HAase, initiating hydrogel degradation through HA cleavage and consequent AIE PSs release (activation phase: “ON” state); (2) Amplification phase: the diffused Ca^2^^+^ (<10 mM) stabilizes HAase conformation, enhancing HAase activity and accelerating AIE PSs release. Subsequent light irradiation generates ROS through activated AIE PSs; (3) Termination phase: pathogen elimination reduces HAase secretion while accumulated Ca^2^^+^ (>10 mM) induces the inactivation of the already existing HAase through salt precipitation, terminating both hydrogel degradation and PSs release (deactivation phase: “OFF” state). This work, for the first time, reports the Ca^2+^‐dependent HAase activity and clarifies its underlying mechanism both experimentally and theoretically. It demonstrates great superiorities of the proposed hydrogel dressing in accelerating the healing of infected wounds in a smarter, safer, and more precise way.

## 2 Results and Discussion

### 2.1 Synthesis and Characterization of AIE PS (TTPy‐NH_2_)

**FIGURE 1:**
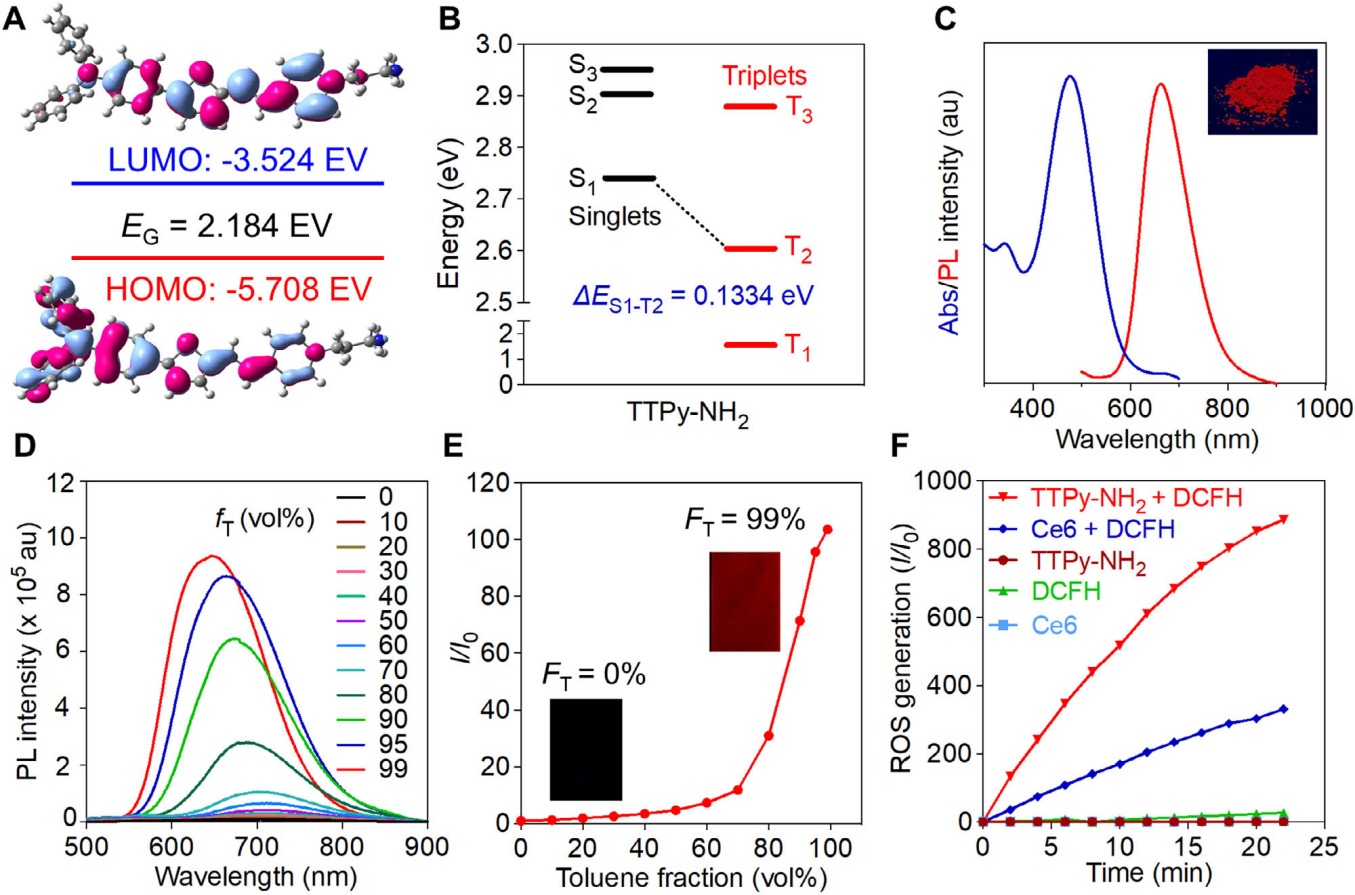
DFT calculation, photophysical and photodynamic properties of TTPy‐NH_2_. (A) Calculated HOMO–LUMO distribution of TTPy‐NH_2_. The narrow bandgap indicates that TTPy‐NH_2_ can absorb visible light (∼568 nm), enabling efficient electron excitation from the HOMO to LUMO under irradiation. (B) The energy levels and Δ*E*_S1‐T2_ value of TTPy‐NH_2_. A significantly reduced Δ*E*_ST_ ​​enhances intersystem crossing and prolongs exciton lifetime​​, synergizing with aggregation‐induced emission to boost ROS generation efficiency for high‐performance antibacterial applications. (C) Absorption spectrum of TTPy‐NH_2_ in water (blue line) and PL spectrum of TTPy‐NH_2_ powder (red line); the inserted picture shows the image of the powder under 365 nm excitation. (D) PL spectra of TTPy‐NH_2_ (10 µM) in a methanol/toluene solvent system with different *f*_T_. (E) Plots of the relative emission intensity (*I/I*_0_) versus *f*_T_, *I*_0_ and *I* are the values of fluorescence intensities of TTPy‐NH_2_ in pure methanol and methanol/toluene mixtures with different *f*_T_. Insert: the corresponding fluorescence images of the samples at the *f*_T_ of 0% and 99%, respectively. (F) ROS generation efficiency of TTPy‐NH_2_ upon visible light irradiation indicated by relative changes in PL intensity of DCFH.

In this work, compound TTPy‐NH_2_ comprising a triphenylamine segment (TPA), a thiophene unit, a carbon‐carbon double bond, and a pyridinium moiety was synthesized as the AIE PSs (Figure S1, Supplementary discussion 1). The TTPA moiety with highly twisted conformation not only serves as a strong electronic donor (D) but also contributes abundant molecular rotors to guarantee the AIE nature. The intensely electron‐deficient pyridinium group is employed as an electronic acceptor (A) and a beneficial segment for water solubility, which is further ensured by the attachment of the propylamine as a hydrophilic tail. The thiophene unit is adopted as an additional D and π‐bridge. Bearing intensive donor‐acceptor (D–A) interaction and extended π‐conjugation [31], TTPy‐NH_2_ is anticipated to be an ideal AIE PS with long‐wavelength fluorescence emission. Moreover, the positively charged pyridinium could also enable the TTPy‐NH_2_ to directly contact negatively charged bacteria, providing an ideal opportunity for the ROS to damage the bacteria [32]. Density functional theory (DFT) calculation showing a small electronic bandgap (*E*_g_) of 2.184 eV and a significantly decreased singlet–triplet energy gap (Δ*E*_ST_) of 0.1334 eV provides the theoretical support for this expectation (Figure 1A, B).

The absorption and photoluminescence (PL) spectra were firstly measured to investigate the photophysical properties of TTPy‐NH_2_. TTPy‐NH_2_ showed an intensive absorption in the visible light region and a satisfactory far‐red/near‐infrared emission that peaked at 663 nm (Figure 1C). To characterize the AIE property, the corresponding PL spectra of TTPy‐NH_2_ in the methanol/toluene solvent system with varied toluene fractions (*f*_T_) were recorded. The PL intensity firstly showed a slight elevation along with the increase of *f*_T_, which further boosted sharply when the *f*_T_ surpassed 70% and finally reached a maximum when the *f*_T_ increased to 99% (Figure 1D, E). The over 100‐fold enhancement of PL intensity and nearly 3‐fold increase of fluorescence lifetime (Figure S2) in the aggregated state compared to that in pure methanol solution undisputedly indicated the typical AIE behavior of TTPy‐NH_2_. Subsequently, the ROS‐generating ability of TTPy‐NH_2_ under visible light irradiation was investigated using dichlorodihydrofluorescein (DCFH) as an indicator. As shown in Figure 1F, the fluorescence intensity of DCFH rapidly enhanced in the presence of TTPy‐NH_2_ along with continuous irradiation, while a negligible increase in fluorescence signal was detected for the irradiated solution with DCFH or TTPy‐NH_2_ alone. In addition, much better overall ROS generation capability was observed in comparison with the commercially available Chlorine 6 (Ce6). These results evidently substantiated that the obtained TTPy‐NH_2_ was a high‐performance AIE PS for the following construction of AIE PS‐loaded hydrogel dressings.

### 2.2 Design and Characterization of Switchable Antibacterial Hydrogel (SAH)

***Design a scenario*** *of SAH*. Inspired by the bacterial infection mechanism that extensive pathogenic bacteria typically secrete HAase to “eat away” the extracellular matrices (e.g., HA) and infect deeper into the tissue [33], HA was firstly selected as the responsive part of the dressing to achieve on‐demand release of PSs based on the degree of infection. Additionally, SA was chosen to assist in gel formation, as it could be easily crosslinked by Ca^2+^ in physiological conditions without catalysts, thereby ensuring the biocompatibility of the prepared hydrogels [34, 35]. More importantly, Ca^2+^ can serve as the regulator of HAase activity. Collectively, by employing both HA and SA as the hydrogel matrices, as well as using Ca^2+^ as a crosslinking agent and HAase activity regulator, a well‐designed SAH allowing Ca^2+^‐dependent HAase responsiveness and on‐demand release of AIE PSs could be obtained.

**FIGURE 2:**
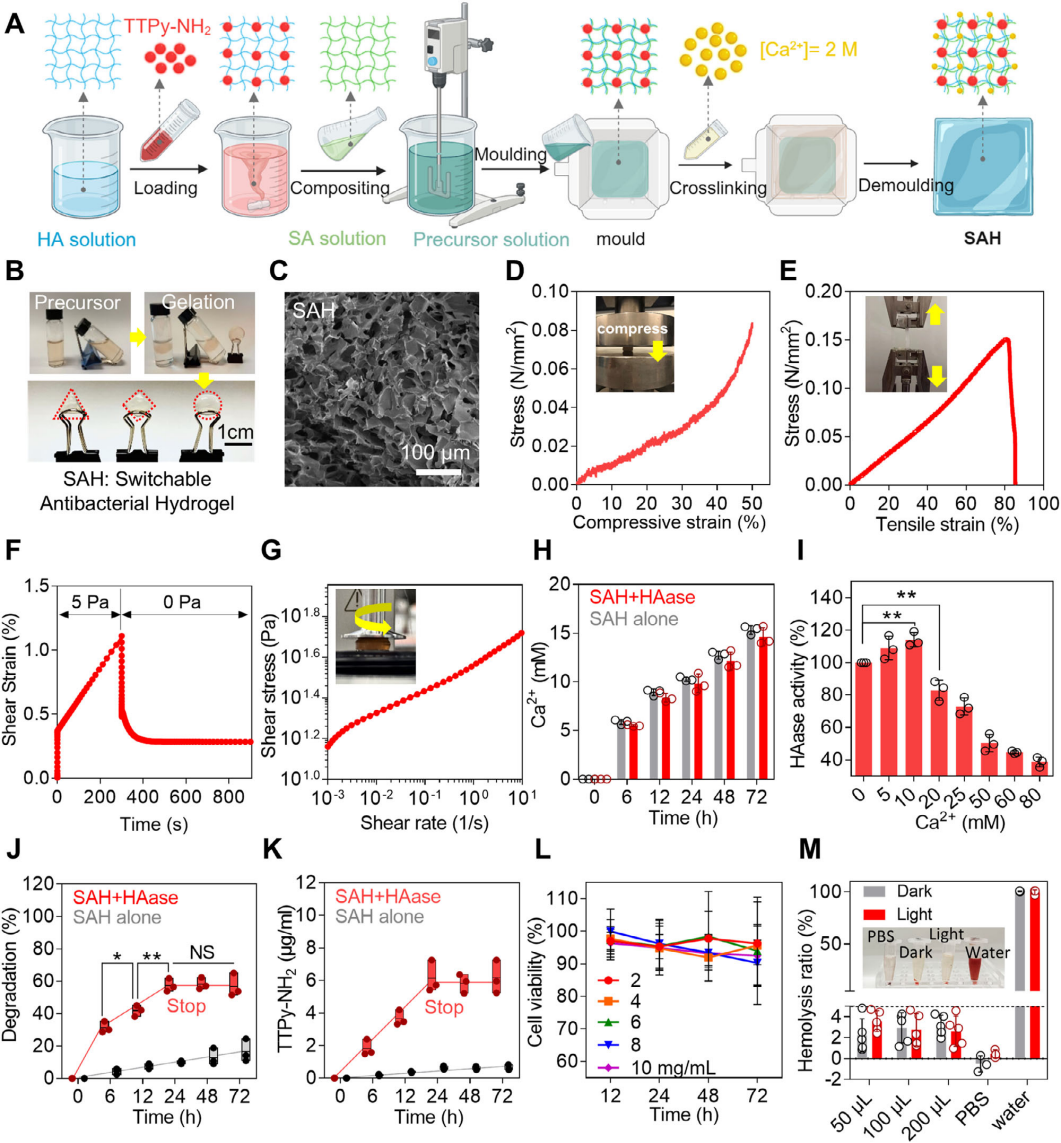
Preparation and Characterization of SAH. (A) The schematic diagram of the preparation of SAH. (B) The gelation process and finished product diagram of SAH: different shapes of SAH can be prepared using different molds. Scale bar: 1 cm. (C) Microstructure of SAH characterized using SEM. Scale bar: 100 µm. (D) Compressive stress‐compressive strain curve; SAH was made into a cylinder (Φ 8 × 8 mm) in a mold. (E) Tensile stress‐tensile strain curve; SAH was made into a plate (2 × 5 × 50 mm) in a mold. (F) The Creep test result was characterized by a rotational rheometer; SAH was made into a cylinder (Φ 150 × 8 mm) in a mold. (G) Stress yield test result characterized by a rotational rheometer; SAH was made into a cylinder (Φ 150 × 8 mm) in a mold. (H) The release of Ca^2+^ from SAH with or without HAase (10 mg/mL). (I) Effect of Ca^2+^ concentration on HAase activity. (J) The degradation of SAH with or without HAase (10 mg/mL). (K) The release of TTPy‐NH_2_ from SAH with or without HAase (10 mg/mL). (L) The cell viability of L929 co‐cultured with SAH upon light irradiation (16 mW/cm^2^) and HAase (2‐10 mg/mL). (M) Hemocompatibility of SAH: The hydrogel was prepared into cylinders with volumes of 50, 100, and 150 µL and incubated with mouse blood cells at a concentration of 4%. *n =* 3, NS: no significance, *p < 0.05, **p < 0.01.

***Physicochemical properties***. For the preparation of SAH, the precursor solution was firstly prepared by incorporating TTPy‐NH_2_ into HA and then mixing with SA (Figure 2A). The first step was to load TTPy‐NH_2_ within the HA. The Fourier transform infrared spectroscopy (FTIR) results in Figure S3A showed that the peaks of TTPy‐NH_2_ at 1485 cm^−1^ (C‐H in‐plane bending vibration) and 815 cm^−1^, 749 cm^−1^ and 689 cm^−1^ (C‐H out‐of‐plane bending vibration) disappeared after the reaction with HA but could still be observed after simply mixing with HA powder. This result indicated that TTPy‐NH_2_ molecules may bind to the HA polymer chain through inter‐ and intra‐molecular hydrogen bonds. The zeta‐potential (Figure S3B) showed that the negative potential of HA remained unchanged before and after the reaction with the positively charged TTPy‐NH_2_, indicating that TTPy‐NH_2_ was successfully loaded into the HA polymer chains rather than simply mixing with HA [36]. The second step was mixing SA and HA as the precursor solution. The FTIR demonstrated that the peaks of SA at 1154 cm^−1^ and 1090 cm^−1^ (‐C = O stretching vibration) [37] were blue‐shifted to 1096 cm^−1^ and 1034 cm^−1^ after the reaction, verifying the existence of electrostatic interaction between SA and HA mediated by carboxyl groups.

The precursor solution was brought into non‐mixing interfacial contact with a 2 M CaCl_2_ solution at a 3:8 volumetric ratio and then crosslinked via the highly concentrated CaCl_2_ to form the final dressing. As shown in Figure S3C, the precursor phase absorbed 0.8 M Ca^2+^ within 20 mins, reaching saturation with no further absorption observed thereafter. Rheological characterization of the precursor solution confirmed its purely viscous liquid state: amplitude sweeps (Figure S3D) and frequency sweeps (Figure S3E) revealed a persistent dominance of loss modulus (G'' > G'), while viscosity curves (Figure S3F) measured a steady‐state viscosity of 1950 mPa·s. In contrast, the Ca^2+^‐crosslinked SAH hydrogel transitioned into a solid‐like material, as demonstrated by a dramatic reversal of moduli dominance [38]. At 1% shear strain (Figure S3G), the elastic modulus (G*'*) plateaued at 10^4^ Pa, surpassing G'' by nearly an order of magnitude, indicative of a tightly crosslinked elastic network [39]. Frequency sweeps (0.1–100 rad/s, Figure S3H) further confirmed structural stability, with frequency‐independent *G'* (∼10^3^ Pa) across the tested range. Stress relaxation tests (Figure S3I) revealed rapid stress decay (relaxation time <1 s) followed by near‐complete recovery, highlighting the hydrogel's ability to dissipate transient strains while maintaining long‐term network integrity [40]. These results collectively underscore the ​​high crosslinking density​​ achieved through diffusion‐mediated Ca^2+^ incorporation, enabling the hydrogel to retain structural fidelity and be molded into diverse geometries (e.g., triangle, square, circle; Figure 2B).

The scanning electron microscopy (SEM) image of SAH (Figure 2C) revealed an intact, interconnected porous microstructure. Pore size distribution analysis demonstrated that 28.0% of pores occupied an area <200 µm^2^, 45.3% fell within 200–1000 µm^2^, and fewer than 2% exceeded 5000 µm^2^ (Figure S3J). This hierarchical porosity optimizes wound exudate management by synergizing capillary action (mediated by smaller pores) and fluid retention (enabled by intermediate‐sized pores), while minimizing oversized cavities (>5000 µm^2^) that could destabilize the structural framework [41]. The resulting architecture not only ensures mechanical stability and efficient oxygen/nutrient diffusion but also validates SAH's efficacy in maintaining fluid homeostasis—critical for accelerating healing in dynamic wound environments. Coupled with its exceptional swelling capacity (Figure S3K), this microstructurally engineered hydrogel exhibits significant potential as a high‐performance dressing by actively absorbing exudates and sustaining a pro‐regenerative wound interface [42]. The mechanical properties of SAH were rigorously characterized to evaluate its suitability as a wound dressing. Compression and tension tests (Figure 2D, E) revealed a compression modulus of 48.5 ± 19.4 kPa and a tensile modulus of 134.5 ± 12.5 kPa, respectively, aligning with the soft yet resilient mechanical profile required for conformable skin adherence and movement accommodation [43]. Creep testing under a constant stress of 5 Pa (Figure 2F) demonstrated exceptional elastic recovery, with minimal transient deformation (<5%) and negligible permanent strain, indicative of dominant viscoelastic behavior rather than irreversible plastic flow [44]. Further corroborating this, yield stress analysis (Figure 2G) showed no detectable flow point (yield stress ∼0 Pa) across shear rates of 0.1–1000 s^−^^1^, confirming the hydrogel's ability to maintain structural integrity under dynamic physiological stresses without yielding. Collectively, these results highlight SAH's optimal balance of softness, elasticity, and stress resistance—critical for ensuring mechanical compatibility with delicate wound tissues while preventing structural collapse during clinical use.

***Release behaviors***. As the regulator of HAase activity, the release of Ca^2+^ from SAH was investigated preliminarily. The concentration of released Ca^2+^ consistently increased throughout the entire tested period, with 10 mM of Ca^2+^ being detected at 24 h. The release profile exhibited a similar trend regardless of the existence of HAase (Figure 2H, SAH + HAase), definitely hinting at the free dissolution of Ca^2+^ from the hydrogel. Further investigation into HAase activity demonstrated that the activity gradually enhanced with the increase of Ca^2+^ concentration, climbing to the maximum when Ca^2+^ reached 10 mM. However, when the Ca^2+^ concentration surpassed 10 mM, the HAase activity was inversely inhibited (Figure 2I). These results provide a reasonable explanation for the following observed profiles of SAH degradation and TTPy‐NH_2_ release. Specifically, during the initial 24 h, when the Ca^2+^ concentration was below 10 mM, both SAH degradation and TTPy‐NH_2_ release gradually enhanced owing to the elevated HAase activity, yielding around 50% degradation of SAH (Figure 2J and Figure S4A) and around 6.2 µg/mL release of TTPy‐NH_2_ (Figure 2J and Figures S4B and S4C) in the presence of 10 mg/mL HAase at 24 h. After 24 h, the Ca^2+^ with high concentration started to deactivate HAase and initiated the “shutting down” machinery of TTPy‐NH_2_ release. Both SAH degradation and TTPy‐NH_2_ release in the hydrogel system reached plateaus at 24 h in the SAH + HAase group; however, the SAH alone without HAase did not follow the above pattern and displayed a continuous but much slower degradation rate (Figures 2J and S4D) and less TTPy‐NH_2_ release (Figure 2K) due to the absence of HAase. Furthermore, we investigated whether hydrogel swelling governs drug release. SAH reached swelling equilibrium within 4 h across all tested liquid environments, with equilibrium swelling ratios of 38.5 ± 6.7% in simulated body fluid (SBF), 46.1 ± 5.7% in PBS, and 67.1 ± 7.7% in ddH_2_O. Regression analysis revealed negligible correlations (all R^2^ < 0.1) between swelling ratios and the release kinetics of the antimicrobial photosensitizer TTPy‐NH_2_, specifically R^2^ = 0.094 (ddH_2_O), 0.095 (SBF), and 0.027 (PBS). These statistically insignificant correlations (R^2^ << 0.25) conclusively demonstrate that swelling‐mediated mass transfer is not a dominant release mechanism under physiological or simulated physiological conditions. These results solidly proved the Ca^2+^‐dependent responsiveness of HAase as well as the HAase activity‐dependent TTPy‐NH_2_ release from the designed SAH.

***Biocompatibility***. We first investigated the biocompatibility of the loaded TTPy‐NH_2_ in dark conditions as well as its phototoxicity under visible light irradiation. Basically, L929 cell, a mouse fibroblast cell line that is typically used in in vitro wound healing studies, was employed as a representative cell model [45]. After co‐culturing with different concentrations (4‐16 µg/mL) of water‐soluble TTPy‐NH_2_, the cell viability of L929 cells in dark conditions remained above 90% as compared with the control group (cell only), indicating that TTPy‐NH_2_ alone had good biocompatibility. Upon exposure to light irradiation, the phototoxicity of TTPy‐NH_2_ was obvious, showing that the cell viability gradually decreased as the concentration of TTPy‐NH_2_ increased (Figure S5A). The cell images captured by optical microscopy also showed that high concentrations of TTPy‐NH_2_ with light irradiation caused bubbles inside the cells and opaque clusters at the cell edges (Figure S5B). These results were in accordance with the literatures, which reported that ROS could increase membrane permeability to cause an unbalanced ionic gradient in cells, finally leading to apoptosis or even necrosis [46, 47]. Briefly, TTPy‐NH_2_ could become phototoxic at high concentrations in the presence of light irradiation, ascribing to its high ROS generation capacity.

We then explored the biocompatibility of SAH, in which the main components were FDA‐approved HA and SA that have been extensively reported to have excellent biocompatibility [48, 49, 50]. Therefore, the biocompatibility research focused on the cytotoxicity of SAH in response to the release of different concentrations of TTPy‐NH_2_ and the consequent production of ROS under light irradiation. When cells were cultured with SAH dressings in the presence of either HAase (2‐10 mg/mL) or light only (16 mW/cm^2^, Figure S5C and S5D), the cell viabilities consistently remained above 90%, indicating the dressings themselves were not toxic to the cells. Notably, the favorable cell viability demonstrated in Figure S5D is consistent with the diminished ROS generation of TTPy‐NH_2_ within the hydrogel (Figure S5E). Both results corroborate the overall design rationale of this study, which posits that TTPy‐NH_2_ exhibits relatively weak ROS generation when encapsulated within the hydrogel matrix. Moreover, the limited amount of ROS generated by TTPy‐NH_2_ is confined to the hydrogel and is not able to diffuse to the targeted sites for PDT. In the presence of HAase, the hydrogel degrades, leading to the release of TTPy‐NH_2_, which will subsequently enhance ROS generation and improve PDT efficacy. However, to our surprise, even when cells were treated with SAH in the presence of both HAase and light, a condition in which ROS generation is enhanced in comparison to that in Figure S5D, over 90% of cell viability can still be observed at different time points (Figure 2L). Microscope analysis also revealed the intact cell structure and satisfactory cell density in all treated groups (Figure S5F). The observed maintenance of high cell viability likely stems from a self‐limiting release mechanism inherent to SAH. While partial release of TTPy‐NH_2_ from the hydrogel initially elevated ROS generation, concomitant continuous Ca^2^^+^ release progressively suppressed further TTPy‐NH_2_ diffusion beyond 24 h (Figure 2K). This Ca^2^^+^‐mediated release termination confined ROS production to levels insufficient to induce cytotoxic photodynamic effects on mammalian cells, thereby preserving cell survival. Importantly, the ROS level—while sublethal to host cells—retains bactericidal efficacy, which could be experimentally validated in follow‐up studies. Such dynamically regulated release profiles collectively highlight the superiority of the on‐demand release of TTPy‐NH_2_ with the shutdown mechanism of SAH in maintaining optimal cell viability. Furthermore, the hemocompatibilities of SAH were systematically evaluated under both light and dark conditions. All tested hydrogel volumes (50, 100, 150 µL) exhibited hemolysis rates below the 5% biocompatibility threshold [51], confirming that neither material volume nor photodynamic activity compromises erythrocyte integrity. These results validate its suitability for direct blood contact in wound care applications.

### 2.3 Exploration of the Regulation Mechanism of Ca^2+^ on HAase

**FIGURE 3:**
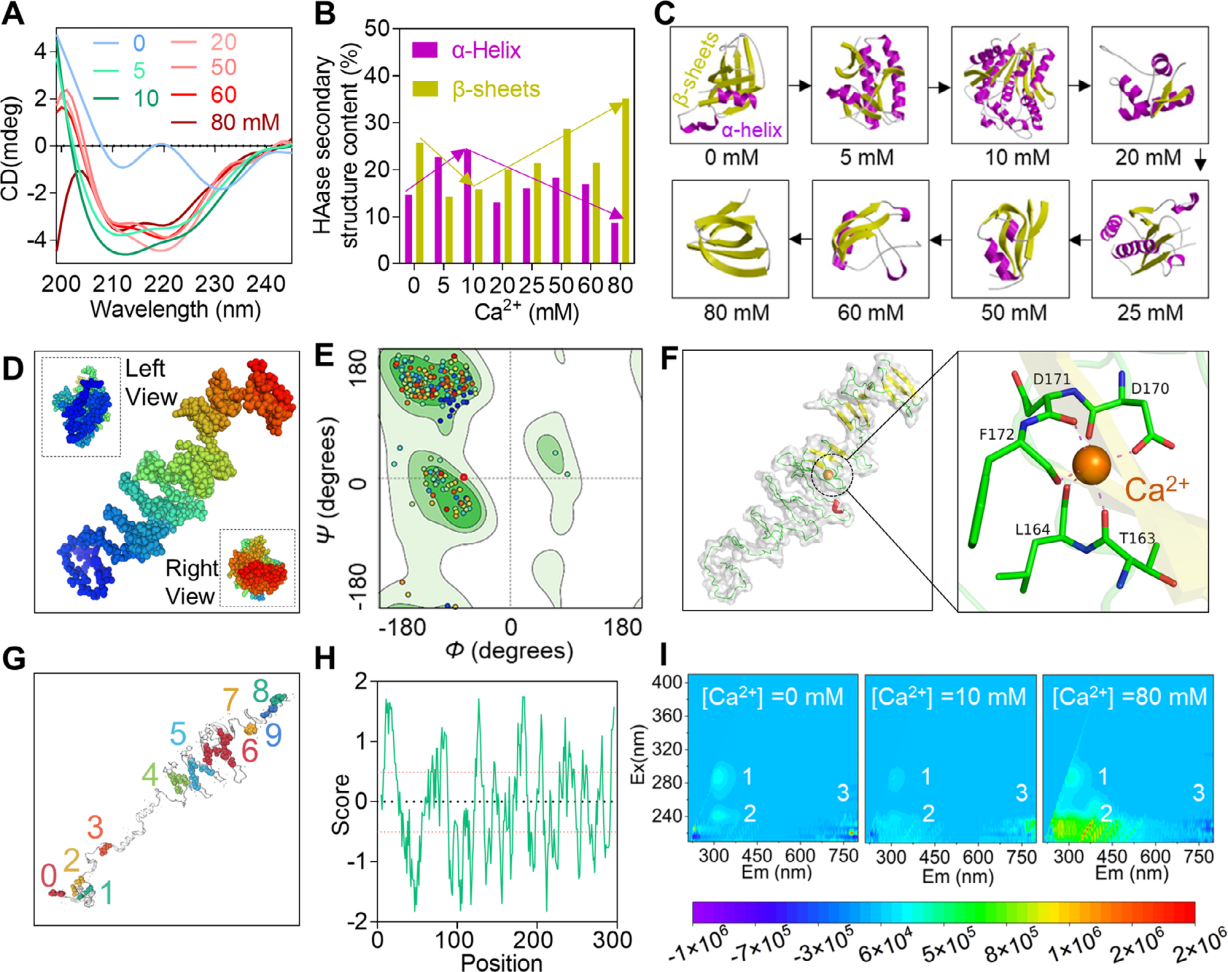
Mechanism exploration of the bidirectional regulation of Ca^2+^ on HAase activity. (A) Circular dichroism spectra of HAase in the presence of Ca^2+^ with different concentrations (0‐80 mM). (B) The estimated secondary structures of HAase with different concentrations of Ca^2+^ (0‐80 mM) calculated by BeStSel software. Arrows indicate changing trends. (C) The estimated representative topologies of HAase with different concentrations of Ca^2+^. The purple showed the α‐helix, and the yellow‐green represented the β‐sheets. (D) The tertiary structure of HAase was constructed by Swiss‐model software. (E) Ramachandran plot of the constructed tertiary structure of HAase: φ denotes the angle of rotation of the C‐N bond to the left of the α‐carbon in a peptide unit, and ψ denotes the angle of rotation of the C‐C bond to the right of the α‐carbon. (F) Molecular docking of HAase with Ca^2+^ by AutoDock software. (G) Hydrophobic clusters in the tertiary structure of HAase (marked by the colored numbers) obtained by the ProteinTools software. (H) Analysis of the hydrophilicity/hydrophobicity of amino acids in HAase obtained by the ProtScale software: negative values indicate hydrophilicity, positive values indicate hydrophobicity, while values between ‐0.5 and +0.5 indicate that amino acids are amphoteric. (I) The three‐dimensional fluorescence maps of HAase with different concentrations of Ca^2+^ (0‐80 mM; groups of other concentrations are shown in Figure S3), T = 298K, and the Rayleigh scattering peaks (Em = Ex) have been removed.

To elucidate the regulation mechanism of Ca^2+^ concentration on the activity of HAase, further exploration was conducted. As a protein, the activity of HAase is generally determined by its secondary structure. Therefore, circular dichroism (CD) was initially used to monitor the secondary structure of HAase upon interaction with different concentrations of Ca^2+^ solutions (Figure 3A). As analyzed by BeStSel software and shown in Figure S6A, the main secondary structures identified in HAase were α‐helix, β‐sheets, and β‐turn, which accounted for more than 61% (Figure S6B, 0 mM Ca^2+^) of the total secondary structures, indicating that HAase was the (α + β) class of protein. Upon exposure to different Ca^2+^ concentrations (0 to 80 mM), the content of α‐helix, regarded as the most stable and rigid secondary structure [52, 53], initially increased before subsequently decreasing. The highest α‐helix content (24.3%) was observed rightly at 10 mM (Figure 3B). Conversely, β‐sheets, as a looser secondary structure, showed the opposite trend. Furthermore, the closest predicted topologies of the Ca^2+^‐treated HAase [54] in Figure 3C further supported the above results. Coincidentally, the proportion of α‐helix positively correlates with HAase activity. Therefore, it can be speculated that Ca^2+^ regulates the enzyme activity by altering the protein stability. Specifically, Ca^2+^ at a concentration below 10 mM could increase the content of α‐helices in HAase and enable the Haase to adopt a more stable conformation, consequently promoting enzyme activity.

Molecular docking studies were further adapted to identify the binding sites between Ca^2+^ and HAase. Firstly, the tertiary structure of HAase was constructed (Figure 3D) and confirmed using the Ramachandran plot in Figure 3E, as all amino acids of HAase are located in the energetically most favorable region. Secondly, Ca^2+^ was employed as a ligand to dock with the constructed HAase structure using the AutoDock Tools software. According to the docking calculation, the binding modes of Ca^2+^ and HAase with the lowest binding energy were shown in Table S2 and Figure 3F. The Ca^2+^ was docked into a helical cavity located in the middle of the HAase and formed a six‐coordination interaction with the O atoms on amino acids Thr (T)‐163, Leu (L)‐164, Asp (D)‐170, D‐171, and Phe (F)‐172 of the protein. Previous studies have revealed that there were hydrogen bonds or electrostatic interactions between the ligand and HAase [55, 56]. This result, for the first time, suggested the existence of coordination bonds between Ca^2+^ and HAase, providing further insight into the capability of Ca^2+^ in enhancing the conformational stability of HAase.

Moreover, upon analysis using ProtScale and ProteinTools software, we discovered numerous hydrophobic amino acids and 10 hydrophobic clusters within the tertiary structure of HAase. According to the previous studies, these clusters are susceptible to exposure and would cause the salting out of HAase through hydrophobic interactions when high concentrations of Ca^2+^ (as a strong electrolyte) bound with H_2_O molecules preferentially within protein [57] (Figure 3G, H, Figure S7 and Table S3). To further validate this, we recorded three‐dimensional fluorescence spectra of HAase at varying concentrations of Ca^2+^ (0‐80 mM). The results indicated that when the Ca^2+^ concentration was below 10 mM, the characteristic peak 1 of HAase was inhibited, and peak 3 was pronounced, indicating the conformational transition of HAase resulted from Ca^2+^. However, when the Ca^2+^ concentration exceeded 10 mM, numerous heterogeneous and broad peaks appeared at peak 2, evidently implying the salt precipitation of protein molecules [58] (Figure 3I and Figure S8).

Collectively, based on the molecular simulations and experimental results, it can be concluded that low concentrations of Ca^2+^ can elevate the activity of HAase by stabilizing its spatial structure. This stabilization is achieved by stimulating the transformation of HAase into its preferential conformation and forming coordination bonds with HAase. Inversely, high concentrations of Ca^2+^ (>10 mM) resulted in the salt precipitation of HAase, leading to its inactivation.

### 2.4 In vitro Antibacterial Activity of SAH

**FIGURE 4:**
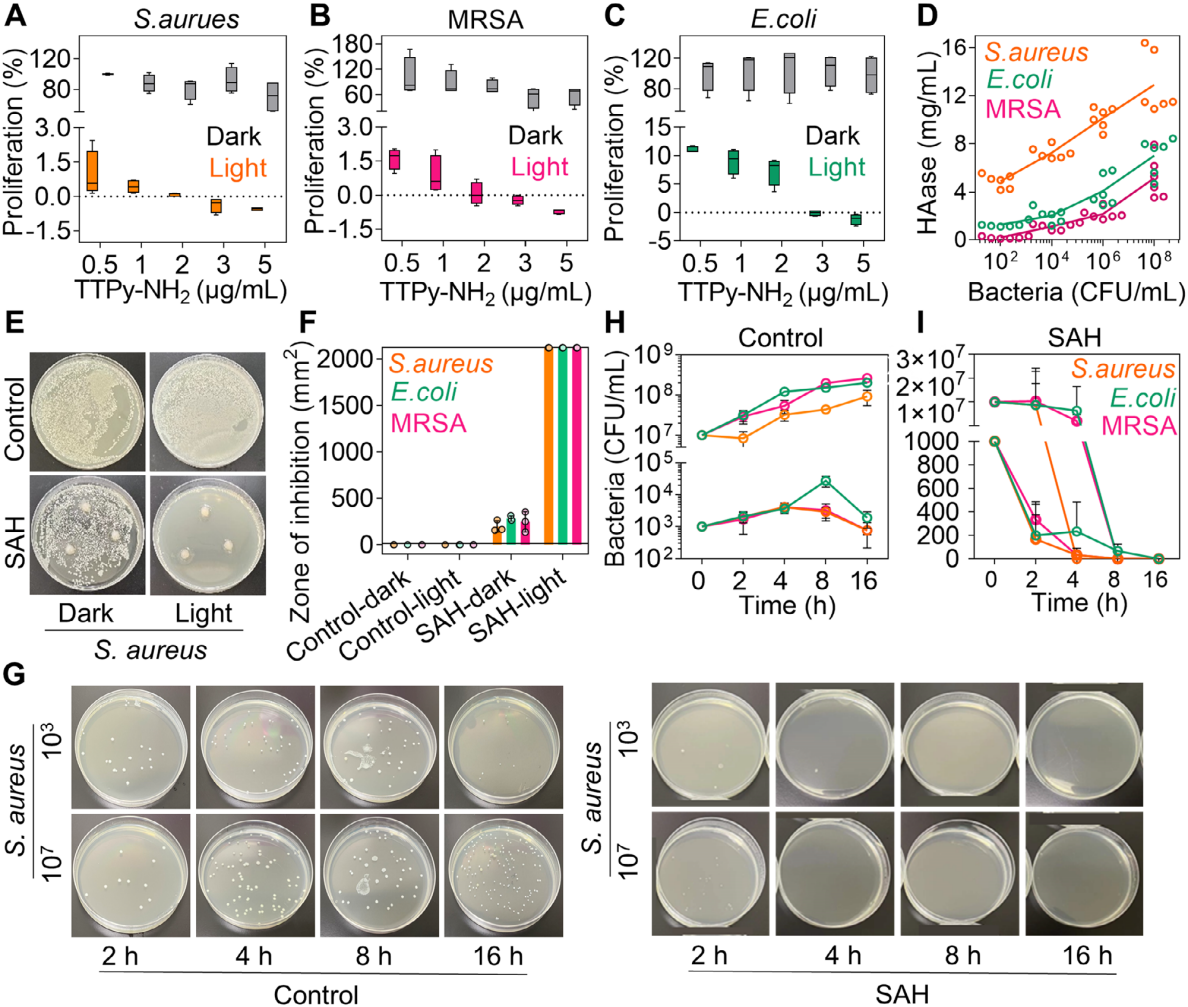
Antibacterial abilities of TTPy‐NH_2_ and SAH. Bacterial proliferation of (A) *S. aureus*, (B) MRSA, and (C) *E. coli* after incubation with TTPy‐NH_2_ at different concentrations (0.5‐5 µg/mL) in dark and light conditions, respectively. (D) The HAase production of three bacteria at different concentrations (10^2^‐10^8^ CFU/mL). (E) The photodynamic antibacterial properties against *S. aureus* of SAH are characterized by inhibition zone assay. (F) The quantitative analysis for the area of the inhibition zone. (G) The antibacterial properties of SAH against *S. aureus* at high (10^7^ CFU/mL) and low (10^3^ CFU/mL) concentrations. Quantitative results of antibacterial properties presented by plate culture for (H) Control, (I) SAH. Hydrogels were firstly incubated with bacteria solution for 2–16 h, and the bacteria solutions were then exposed to white light irradiation (16 mW/cm^2^) for 20 mins.

Prior to the assessment of antibacterial activity, the strong binding affinity of TTPy‐NH_2_ to *Escherichia coli* (*E. coli*, a representative of gram‐negative bacteria), *Staphylococcus aureus* (*S. aureus*, a representative of gram‐positive bacteria), and methicillin‐resistant *Staphylococcus aureus* (MRSA, a representative of multidrug‐resistant bacteria) was confirmed. As illustrated in Figure S9, after a 15 min incubation with TTPy‐NH_2_, all of the above three bacteria emitted bright red fluorescence, indicating that TTPy‐NH_2_ exhibited a strong binding affinity to the bacteria. This adequate contact between TTPy‐NH_2_ and bacteria is essential for effective photodynamic antibiosis. Subsequently, the antibacterial efficiency of TTPy‐NH_2_ was explored. The bacterial proliferation of these species after incubating with TTPy‐NH_2_ at various concentrations (0.5‐5 µg/mL) in both dark and light conditions is shown in Figure 4A–C. When the concentration of TTPy‐NH_2_ reached 2 µg/mL, the reproduction of *S. aureus* and MRSA was completely inhibited. For *E. coli*, a concentration of TTPy‐NH_2_ above 3 µg/mL was required for complete inhibition. Control groups maintained in the dark exhibited no obvious bactericidal activity. These results evidently proved the antibacterial efficiency of TTPy‐NH_2_. Furthermore, as a key factor for TTPy‐NH_2_ releasing, the productivity of HAase by all bacteria was evaluated. As shown in Figure 4D, the concentration of HAase secreted by all bacteria was positively correlated with their concentration. Even at low bacterial concentration (10^2^ CFU/mL), the concentration of secreted HAase was sufficient to reach the required levels necessary to trigger the release of TTPy‐NH_2_ (Figure S4).

These results established a robust foundation for the excellent antibacterial performance of SAH, as validated by the inhibition zone assay, which showed complete elimination of the entire cultures with just three drops of hydrogels (Figure 4E, F and Figure S10A). Additionally, to explore whether the hydrogel can release TTPy‐NH_2_ in the presence of different concentrations of bacteria to achieve the bactericidal effect, we tested the photodynamic antibacterial efficiencies of SAH in the presence of both high (10^7^ CFU/mL) and low (10^3^ CFU/mL) concentrations of bacteria (Figure 4G–I). The reason for choosing 10^3^ CFU/mL as the low bacterial concentration was that the World Health Organization (WHO) standards claimed that a bacterial concentration above 10^3^ CFU/mL was usually regarded as the concentration for an infection [59]. SAH was able to completely kill all bacteria within 16 h when exposed to three types of bacteria with different concentrations (Figure 4G and Figure S10B). Specifically, at low bacteria concentration, SAH still could release sufficient TTPy‐NH_2_ to completely kill all three types of bacteria in 4 h. However, when the bacteria concentration was high, a longer time (8 h) was needed to accumulate more released TTPy‐NH_2_ to clear MRSA. Overall, the prepared dressings exhibited excellent antibacterial effects. (Figure 4G–I and Figure S10B).

### 2.5 in vivo Wound Healing

**FIGURE 5:**
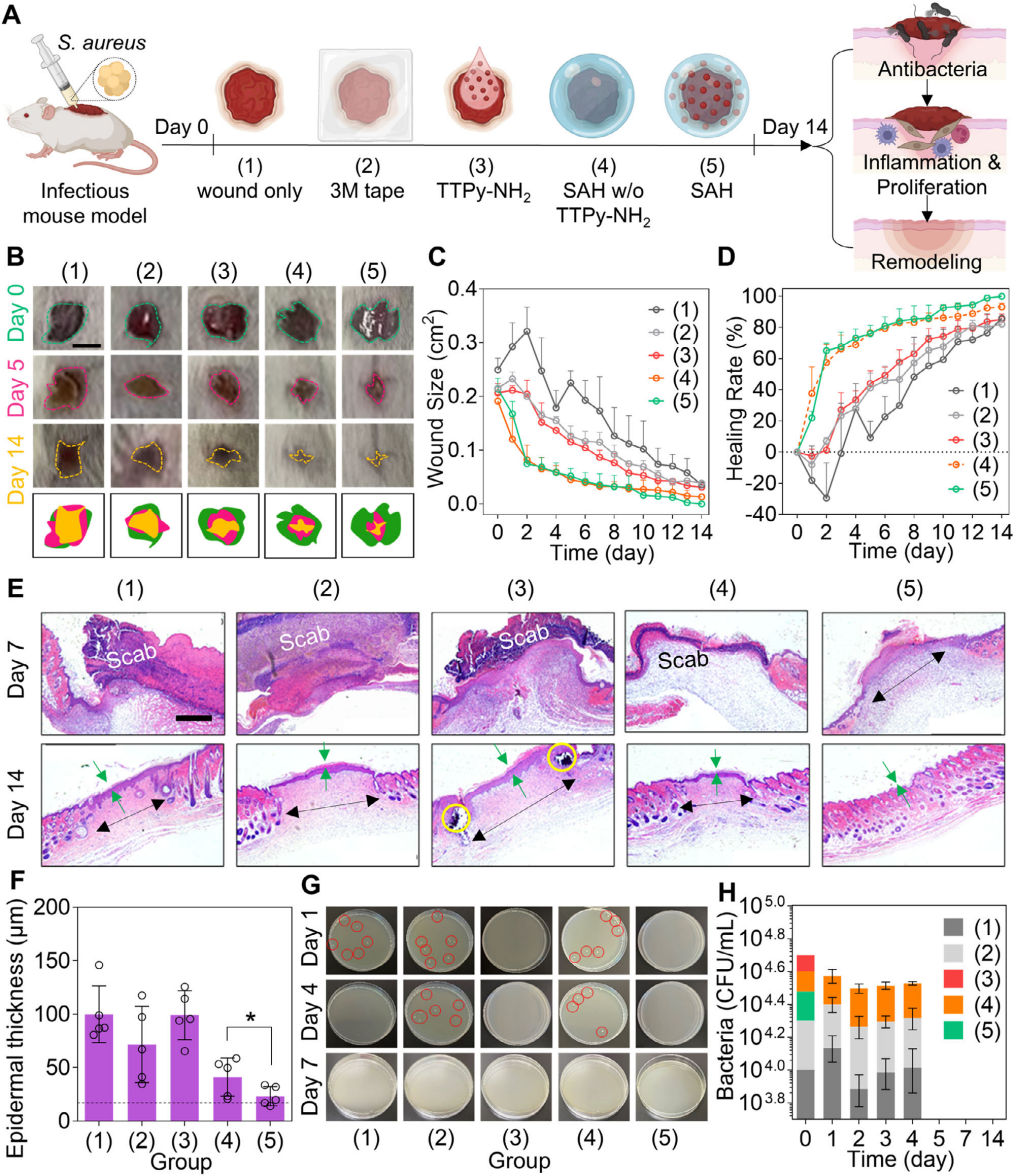
In vivo healing effect of the infected wounds. (A) The design and process scheme of the animal experiment. (B) Representative pictures of the wound sites and the traces of wound‐bed closure in different groups on days 0, 5, and 14, scale bar: 5 mm. (C) Wound size over time in mice treated with different groups. (D) Wound healing rates for different groups during the treatment. (E) Hematoxylin and eosin (H&E) staining of the wound tissue slices for different groups on days 7 and 14. Black double arrows indicate the length of the wound. Green arrows indicate the epidermal layer. The yellow circle indicates the focal calcification. Scale bar: 500 µm. (F) Quantification of the epidermal thickness of samples collected on day 14 (*n =* 5, *p < 0.05). The black dotted line indicates the epidermal thickness of healthy skin. (G) The plate culture images of bacteria sampled from the tissue fluid at wound sites under different treatments on days 1, 4, and 7. Red circles indicate bacteria colonies. (H) The counted number of colonies from the plates in G.

A full‐thickness cutaneous infected wound model was established to evaluate the efficiency of SAH in promoting wound healing [60]. The experimental groups (Figure 5A) were set as follows: wound only, 3M tape (wounds covered with commercial tapes), TTPy‐NH_2_, SAH w/o TTPy‐NH_2_ (hydrogel only), and SAH (TTPy‐NH_2_‐loaded hydrogel). The hydrogel wound dressing was secured to the wound site using 3 M tape to prevent displacement or gnawing by the mice (Figure S11A). Photographic documentation of the wound site is performed, as illustrated in Figure S11B, to ensure the accuracy of the records. All groups received light irradiation. As the images shown in Figures 5B and Figure S11C and S11D, the wound‐only group appeared to tear along with suppuration, indicating severe infection. Wounds in the 3M tape group and TTPy‐NH_2_ group displayed improvements in healing compared with the wound‐only group. For the SAH w/o TTPy‐NH_2_ group and the SAH group, no yellow pus developed in the wound area during the treatment period, and the wounds were kept moist until they formed crusts. In addition, the wound size during the treatment was calculated and displayed in Figure 5C, followed by the determination of the healing rate (Figure 5D). As for the healing rate, two hydrogel groups exhibited faster healing compared with the other three groups, and the SAH group was the first to achieve a complete wound healing rate.

Furthermore, hematoxylin and eosin (H&E) staining revealed significant scabbing in the wound only, 3M tape, and TTPy‐NH_2_ groups, while the SAH w/o TTPy‐NH_2_ group displayed small scabs, and the SAH group had negligible scabs on day 7. By day 14, only the SAH group showed significant healing and possessed the most complete regenerative skin accessories, such as hair follicles (Figure 5E). Notably, the TTPy‐NH_2_ group exhibited extensive tissue necrosis and focal calcification (Figure 5E, yellow circle), particularly in the adjacent connective tissues, implying that excessive ROS may hinder the normal healing process. The regenerated epidermal layer (Figure 5E, green arrows) in the SAH group exhibited greater uniformity compared to the other groups, with a corresponding thickness measured to be 23.3 ± 8.0 µm, which was closest to that of healthy skin (Figure 5F, black dotted line), indicating superior skin re‐epithelialization in the SAH group.

Infectious wound healing is accompanied by a complex biological process including antibacterial effect, inflammation, proliferation, and remodeling [61]. Therefore, the wound healing in different groups was characterized by these aspects. Specifically, for antibacterial effect, the tissue fluids from the wound sites were collected and incubated in Luria‐Bertani liquid medium for 12 h, followed by subsequent plate culture for another 12 h [62]. As shown in Figures 5G, H and Figure S12, the bacteria in the group treated with SAH were completely eradicated on day 1. However, it took 4 days for the SAH w/o TTPy‐NH_2_ group to eliminate the bacteria. Likewise, when TTPy‐NH_2_ was added directly to the wounded tissue, although it killed all bacteria on day 1, it seems not as effective as the SAH group.

**FIGURE 6:**
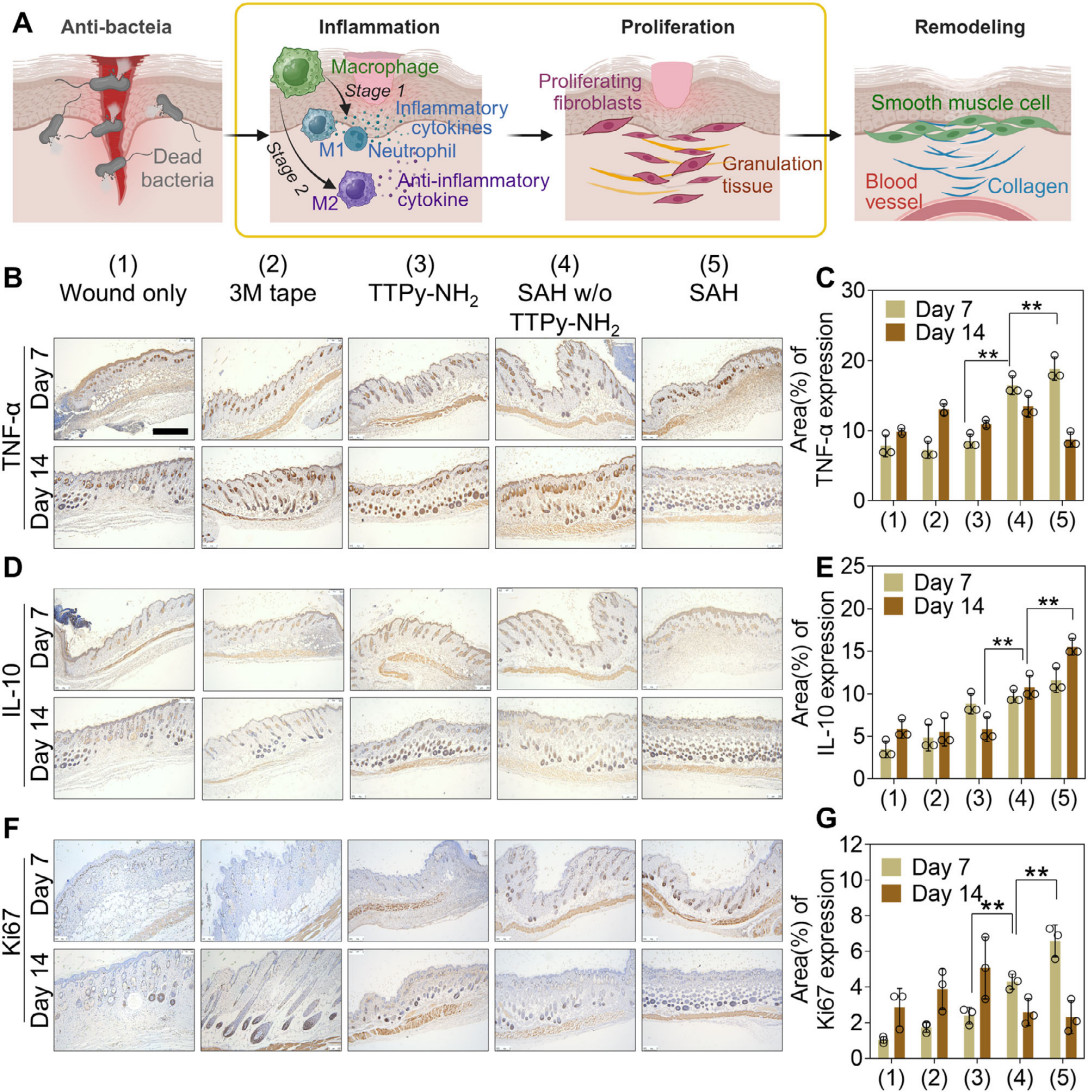
Assessment of the inflammation and proliferation processes in wound healing. (A) Typical process for the healing of an infected wound. This section focuses on the inflammation and proliferation (yellow box). (B–E) The characterization of inflammation by immunohistochemical staining of (B) TNF‐α and (D) IL‐10 for different groups on days 7 and 14. Quantitative analysis of (C) TNF‐α and (E) IL‐10 expression for different groups on days 7 and 14 (*n =* 3, **p < 0.01). (F) The characterization of proliferation by immunohistochemical staining of Ki67. (G) Quantitative analysis of Ki67 expression for different groups on days 7 and 14 (*n =* 3, **p < 0.01). Scale bar: 500 µm.

The inflammatory phase of wound healing contributes to the removal of damaged cells and the recruitment of tissue repair cells, which is subsequently followed by a transition to an anti‐inflammatory phase that facilitates tissue repair (Figure 6A) [63]. To evaluate the inflammation‐modulating effects of SAH dressing, the expression of inflammatory indicators throughout the treatment was assessed histologically. Tumor necrosis factor alpha (TNF‐α) is a representative factor involved in the inflammatory response phase of wound healing [64]. The expression level of TNF‐α in the SAH group was statistically higher than other groups (SAH w/o TTPy‐NH_2_: *p* = 0.004, TTPy‐NH_2_: *p* = 0.0007, 3 M type: *p* = 0.0002, Wound only: *p* = 0.00002) on day 7 (Figure 6B, C), indicating that more neutrophils and monocytes were recruited to the wound site and differentiated into inflammatory phenotype macrophages (M1). Subsequently, on day 14, TNF‐α expression in the SAH group was significantly down‐regulated, which is conducive to wound healing [64]. Meanwhile, as a key anti‐inflammatory cytokine promoting the regenerative phase during wound healing [65], interleukin‐10 (IL‐10) was more highly expressed in the SAH group on day 14 than that observed in other groups (Figure 6D, E). Changes in the inflammatory environment from day 7 to day 14 indicated that wounds had transitioned to the proliferation phase on day 14 [63]. The cell proliferation activity during granulation tissue formation was further assessed using Ki67 immunostaining. The expression of Ki67 in the SAH group was significantly higher on day 7 but markedly lower on day 14 compared to all other groups (Figures 6F, G), indicating that the wound progressed from a proliferative stage to a remodeling stage. Moreover, the decreased Ki67 on day 14 also suggested the inhibition of the abnormal tissue proliferation [61].

**FIGURE 7:**
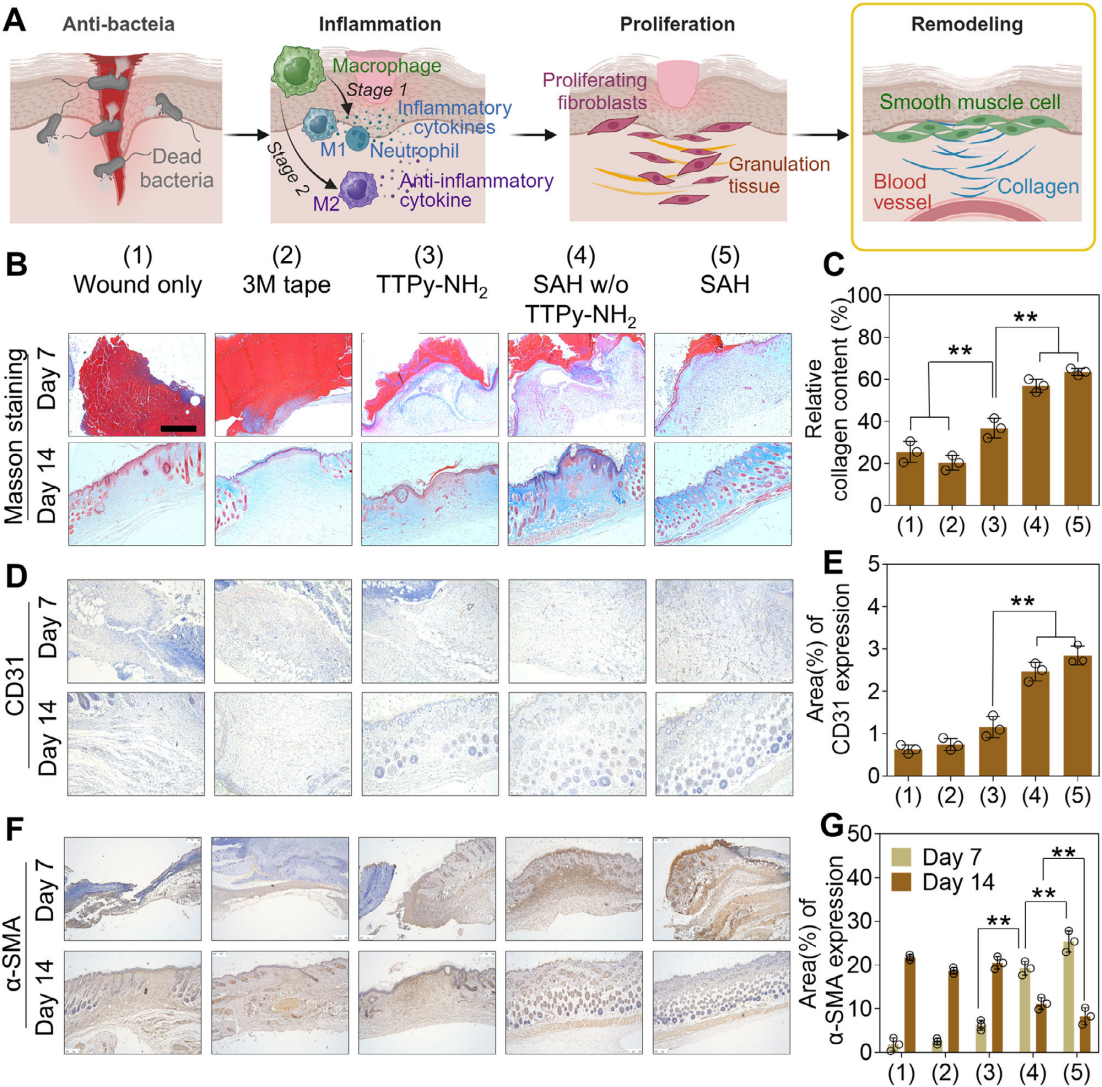
Assessment of the remodeling process in wound healing. (A) Typical process for the healing of infected wound healing. This section focuses on the remodeling (yellow box). (B) Masson's trichrome‐stained slices of wound tissue from different groups on days 7 and 14. (C) Quantitative analysis of collagen content in wound tissue for different groups on day 14 (*n =* 3, **p < 0.01). Immunohistochemical staining of (D) CD31 and (F) α‐SMA in wound tissue for different groups on days 7 and 14. Quantitative analysis of (E) CD31 and (G) α‐SMA expression of each group on day 14 (*n =* 3, **p < 0.01). Scale bar: 500 µm.

We then assessed the final phase of wound healing, known as remodeling, which involves collagen deposition, angiogenesis, and skin regeneration (Figure 7A). Collagen is a key component of dermal tissue and crucial for healing [61]. Therefore, the formation of collagen in the wound tissue was assessed using Masson's trichrome staining (Figure 7B, C). On day 14, collagen deposition was significantly higher in two hydrogel groups (groups 4 and 5) compared to the TTPy‐NH_2_ group (*p* = 0.007 and *p* = 0.01, respectively). The expression of platelet‐endothelial cell adhesion molecule 1 (CD31), as a typical marker for assessing vascularization [64], was significantly elevated in the SAH group as compared with the other groups on day 14 (Figure 7D, E). These results, along with the morphological characterization of neovascularization (Figure S13), demonstrated that SAH was superior in promoting angiogenesis in infected wounds. During wound healing, the activated fibroblasts, which are essential component cells of neo‐formed connective tissue, differentiate into myofibroblasts marked by the neo‐expression of alpha‐smooth muscle actin (α‐SMA) [66]. Typically, granulation tissue begins to form approximately one week post‐wounding, as indicated by the presence of α‐SMA. We analyzed the expression of α‐SMA (Figure 7F, G), in which high levels were observed in groups 4 and 5 on day 7, with the most pronounced expression in the SAH group, indicating a faster wound closure. Besides, on day 14, α‐SMA levels in the SAH group decreased significantly compared to those in other groups, which could be due to the reduced granulation tissues formation in the SAH group, facilitating wound remodeling [66].

Overall, SAH demonstrated exceptional efficacy in promoting wound healing by virtue of its favorable abilities in enabling the on‐demand release of TTPy‐NH_2_. Additionally, it rationally modulates both inflammation and anti‐inflammation processes as well as accelerates cell proliferation and remodeling phases of the wound.

## 3 Conclusion

In summary, we have successfully designed a novel antibacterial hydrogel with the capability of releasing AIE PSs on‐demand via a Ca^2+^‐dependent HAase responsive mechanism for the treatment of infected wounds. The wound dressing was fabricated from the incorporation of antibacterial TTPy‐NH_2_ into polymeric hydrogel networks composed of HA and SA upon crosslinking in Ca^2+^ solution. When exposed to Ca^2+^ with a concentration below 10 mM, HAase tends to adopt a more stable conformation with increased α‐helix content and form coordination bonds with Ca^2+^, ultimately promoting the activity of HAase. However, Ca^2+^ concentration exceeding 10 mM would lead to the salt precipitation and inactivation of the HAase. The Ca^2+^‐dependent HAase responsiveness endowed the resulting SAH with the advantageous capacity of on‐demand release of TTPy‐NH_2_, which can not only enable the sufficient AIE PSs release for bacteria clearance in the initial phase but also timely initiate the shutting down mechanism to prevent unnecessary cytotoxicity and damage from superfluous ROS. With all these beneficial features, SAH demonstrated excellent performance in promoting the wound healing in the full‐thickness cutaneous wound model with *S. aureus* infection. It is worth mentioning that this work, for the first time, reported the Ca^2+^‐dependent responsiveness of HAase and further elucidated the corresponding regulation mechanisms by virtue of molecular modeling and spectroscopic analysis. Additionally, we believe that the developed hydrogel dressing holds great potential as the next generation of ROS‐based antibacterial wound dressings in clinical settings.

## Conflicts of Interest

The authors declare the following financial interests/personal relationships, which may be considered as potential competing interests:

Danyang Li, Rongwei Cui, Lizhou Xu, Zhijun Zhang, and Miaomiao Kang have a patent licensed to The Seventh Affiliated Hospital of Sun Yat‐sen University.

## Supporting information




**Supporting File 1**: exp270140‐sup‐0001‐SuppMat.docx.

## Data Availability

Data will be made available on request.
