# Supplementary material for: Antibacterial Hydrogel Dressing With Ca2+‐Dependent Hyaluronidase Responsiveness for Accelerating Wound Healing via On‐Demand Release of AIE Photosensitizers
Source: Exploration (Beijing). 2026 Feb 26:20250037. Online ahead of print. doi: 10.1002/EXP.20250037 (PMC13393510; doi:10.1002/EXP.20250037)
Supplement: Supplementary file 1 — Supporting File 1: exp270140‐sup‐0001‐SuppMat.docx. [file EXP2-9999-0-s001.docx]

Supporting Information

**Antibacterial Hydrogel Dressing with Ca^2+^-Dependent Hyaluronidase Responsiveness for Accelerating Wound Healing *via* On-Demand Release of AIE Photosensitizers**

**Experiment section**

**Materials**

2', 7'-dichlorodihydrofluorescein diacetate (DCFH-DA) and sodium alginate (Brookfield viscosity ≥ 2000 cps) were purchased from Sigma-Aldrich. Chlorin e6 (Ce6) was obtained from J&K. Phosphate buffered saline (PBS, pH 7.4) was purchased from Thermo Fisher. Hyaluronic acid (1500-2500 kDa) and hyaluronidase (HAase, ≥300 U/mg, from *Steptomyces Hyalurolyticus*) freeze-dried powder were purchased from Mackin. CaCl_2_ (≥96.0%) was purchased from Aladdin. 3,5-dinitrosalicylic acid (DNS) reagent was purchased from Solarbio. Horse serum, Minimum Essential Medium (MEM), penicillin/streptomycin, Luria-Bertani (LB) broth and agar were purchased from Gibco. Alamar blue reagent and 4% paraformaldehyde were purchased from warbio. All chemical agents and solvents were used as received without further purification. The mouse fibroblast cell, L929 cell, was purchased from Biospecies. The bacteria, *Escherichia coli* (*E. coli*, ATCC® 25922™), *Staphylococcus aureus* (*S. aureus*, ATCC® 25923™) and Methicillin-resistant *Staphylococcus aureus* (MRSA, ATCC® 43300™) were gifts from Dr. Xiao-Yong Zhan.

**Synthesis of TTPy-NH_2_**

The TTPy-NH_2_ was synthesized according to the literature (Fig. S1a)^[1]^.

**Characterization of the TTPy-NH_2_ molecule**

^1^H NMR and ^13^C NMR data were recorded on a NMR spectrometer (Bruker AVANCE III, Bruker, switzerland) with MeOD and DMSO-d_6_ as the solvents, respectively. High-resolution mass spectrum (HRMS) was performed on a mass spectrometer (Xevo G2-XS Qtof, Waters, USA) operating in a MALDI-TOF mode. Absorption spectra were measured on a spectrophotometer (Lambda 950, PerkinElmer, USA). Photoluminescence (PL) spectra were recorded on a fluorescence spectrophotometer (FS5, Edinburgh instruments, UK). Fluorescence quantum yields were measured using an absolute fluorescence quantum yield (QY) spectrometer (C11347 Quantaurus QY, Hamamatsu, Japan).

**ROS detection of TTPy-NH_2_ in aqueous solution**

A commonly used ROS indicator DCFH-DA was utilized to detect the ROS generation of TTPy-NH_2_ and Ce6 (as positive control) in aqueous solution under white light irradiation (16 mW/cm^2^). Briefly, 0.5 mL of DCFH-DA in ethanol (1 mM) was added to 2 mL 0.01 M NaOH and allowed to stir under the dark condition for 30 mins. Then the hydrolysate was neutralized with 10 mL of PBS at pH 7.4 and kept in dark before use. By the time, DCFH-DA was hydrolyzed to DCFH. Then the ROS indicator (40 μM) in PBS was further diluted to 5 μM in the sample solution of TTPy-NH_2_ or Ce6 (1 μM) for measurement by PL instrument (FS5 fluorescence spectrophotometer, Edinburgh, UK). The fluorescence of 2', 7'-dichlorofluorescein triggered by TTPy-NH_2_/Ce6-sensitized ROS under white light irradiation was measured at different time intervals. The PL spectra were measured with excitation at 485 nm and emission was collected from 500 to 620 nm. The fluorescence intensity at 525 nm was recorded to indicate the generation capacity of ROS.

**Preparation of switchable antibacterial hydrogel (SAH)**

SAH dressing was prepared by sol-gel method^[2]^. The reaction route is shown in **Scheme 1**. Briefly, AIE PS solution (TTPy-NH_2,_ 0.25 mg/mL), hyaluronic acid solution (HA, 1500-2500 kDa, Macklin, 2 mg/mL) and sodium alginate solution (SA, Brookfield viscosity ≥ 2000 cps, Sigma-Aldrich, 6.0 wt.%) were prepared respectively. The TTPy-NH_2_ solution and HA solution were mixed at a volume ratio of 1:10 and stirred magnetically in a water bath at 40°C until it was homogeneous and clear. The precursor solution was obtained by mixing the above prepared solution with SA solution at a ratio of 11:20 and stirred magnetically at 40°C in a water bath until the whole system did not delaminate. The bubbles generated by stirring during the preparation of precursor solution could be removed by standing at 4 ^o^C overnight. The precursor solution was placed in a six-well plate (or other mould), and CaCl_2_ (≥96.0%, Aladdin) solution at a concentration of 2 M was slowly added along the wall of the plate to an excess (in this study, the volume ratio of precursor solution: CaCl_2_ was 3:8) and left to stand for 2 h at room temperature to obtain SAH. We used the inductively coupled plasma spectrometer (Optima 7000DV, Perkin Elmer, USA) to detect the concentration of CaCl_2_ solution (Ca^2+^) after the reaction to determine the concentration of Ca^2+^ in SAH. The specific concentrations of each component in the SAH are summarized in the table below.

**Table S1**. Composition and Quantities of Hydrogel Components.

| **Component** | **Initial concentration** | **Volume** | **Final**  **concentration** |
| --- | --- | --- | --- |
| HA | 2 mg/ml | 10 | 0.65 mg/mL |
| SA | 6 wt.% | 20 | 38.71 mg/mL |
| AIE | 0.25 mg/ml | 1 | 14.18 μmol/L |
| Ca^2+^ | 2 mol/L | 0 | 2.35 mmol/mL |

**Characterisation of the SAH**

**Zeta potential**

The change of surface charge before and after the reaction between the components was characterized. Samples were prepared by dispersing 50 µL solutions in 1.5 mL of 10 mM PBS at RT. The zeta potential for samples was measured using Zetasizer Nano (Malvern Panalytical, UK) at RT, in triplicates and repeated for three independent batches.

**Fourier transform infrared spectroscopy (FT-IR)**

Fourier transform infrared analyzer (5700, Nicolet, USA) was employed to characterize the changes of chemical groups during the preparation of SAH. All samples were freeze-dried (Lyoquest-85, Telstar Technologies, Spain) at -80 °C for at least 24 h and ground into powder then mixed grinding and tableting with KBr at a mass ratio of 1:100, respectively. The infrared spectrum was recorded ranging from 400 to 4,000 cm^-1^ in wave number.

**Morphology by scanning electronic microscopy (SEM)**

The morphology of SAH was observed by scanning electronic microscopy (SEM, JSM 7800F, Jeol, Japan). The sample was frozen in liquid nitrogen and lyophilized in freeze dryer (Lyoquest-85, Telstar Technologies, Spain) for 24 h. Prior to SEM analysis, dried samples were sputter-coated with a layer of gold (EM ACE600, Leica, Germany).

**Mechanical properties of SAH**

Compressive testing of SAH cylinders (Φ 8 × 8 mm) was determined by using a universal mechanical machine (AGS-X-10kN, Shimadzu, Japan) on the compression rate of 0.5 mm/min until reaching the degree of compression as 80%. The compressive stiffness was calculated based on the linear deformation region of the stress-strain curve from 5% to 15% of compressive strain^[3]^. Tensile strengths and Young's moduli were measured by a universal testing instrument (AGS-X-50N, Shimadzu, Japan) for SAH plates samples (2 × 5 × 50 mm) at a crosshead speed of 1 mm/min with a gage length of 30 mm.

**Rheological characterization**

The rheological properties of the hydrogel precursor and crosslinked hydrogel were evaluated using a rotational rheometer (TA Instruments DHR-3) with a 20 mm parallel plate geometry at a controlled temperature of 25°C. For the precursor solution, amplitude sweep tests were conducted by logarithmically varying the shear strain from 0.01% to 100% at a fixed frequency of 1 Hz to identify the linear viscoelastic region. Frequency sweep tests were then performed within this linear region, spanning angular frequencies from 0.1 to 100 rad/s at a constant strain of 1%. The steady-state viscosity profile was measured by applying shear rates from 0.01 to 100 s⁻¹.

For the crosslinked hydrogel, amplitude and frequency sweep tests were repeated under identical strain and frequency ranges to assess structural stability. Stress relaxation behavior was characterized by applying a 1% instantaneous strain and monitoring the shear stress decay over 600 s. Creep compliance was evaluated under a constant stress of 5 Pa for 300 s, followed by stress removal to quantify strain recovery. The yield stress was determined by incrementally increasing the applied stress from 0.1 to 1000 Pa while tracking the critical stress threshold corresponding to network failure^[4]^.

**Swelling properties of SAH**

The swelling properties of SAH was characterized by weighing method. Briefly, the initial weight (W_0_) of the SAH was measured after being freeze-dried (Lyoquest-85, Telstar Technologies, Spain). Then, the hydrogel samples (n = 5) were immersed in a PBS buffer (pH = 7.4) at 25 °C until the equilibrium state. The swollen hydrogels were weighed (W_t_) after gently removing excess water using filter paper. The swelling ratio of hydrogel can be calculated by the following Equation:

$\text{Swelling ratio \%}\text{ }\text{=}\frac{{(W}_{t}{-W}_{0})}{W_{0}}\text{×}\text{ }\text{100 \%}$ (1)

**The Ca^2+^ release kinetics of SAH**

The Ca^2+^ release kinetics of SAH with or without HAase were both explored. For groups without HAase, SAH were incubated in an equal volume (1 mL) of deionized water immediately after preparation at 37℃. For groups with HAase, SAH were incubated in an equal volume (1 mL) of HAase solution (2 mg/mL) at 37℃. The incubation solution was taken at intervals (0, 6, 12, 24, 48 and 72 h) to detect the concentration of Ca^2+^ using the inductively coupled plasma spectrometer (Optima 7000DV, Perkin Elmer, USA).

**The degradation of SAH**

Two distinct methodologies were employed to characterize the degradation of SAH hydrogel, each addressing a specific mechanistic aspect. First, the 3,5-dinitrosalicylic acid (DNS) assay was utilized to selectively quantify the enzymatic degradation of the hyaluronic acid (HA) component by bacterial-secreted hyaluronidase (HAase). This approach directly correlates HA degradation with the triggered release of the antimicrobial photosensitizer TTPy-NH₂, as HA cleavage disrupts the hydrogel network to liberate encapsulated agents. Second, a gravimetric method was applied to assess the hydrogel’s bulk degradation by measuring mass loss over time. This method evaluates the cumulative material disintegration regardless of enzymatic specificity, thereby providing complementary insights into the hydrogel’s structural stability under physiological conditions^[5]^.

***DNS Assay:*** For assessing the degradation of the hydrogel wound dressing, SAH (1 mL) was immersed in deionized water (as control group) or a medium concentration of 2 to 10 mg/mL HAase solution (as experimental group) at a volume ratio of 1:1 at 37℃. The incubation solution was taken at intervals (0, 6, 12, 24, 48 and 72 h) to detect the concentration of reducing sugar produced by HA by DNS chromogenic method as described above. When the concentration of reducing sugar in the incubation medium no longer increased with the increase of incubation time, it was regarded as the termination of reaction that HA HAase was completely degraded by HAase. The degree of degradation of SAH was calculated by the following Equation:

$\text{The degree of degradation \% = }\frac{\text{A}_{\text{experimental group}}\text{-}\text{A}_{\text{control group}}}{\text{A}_{\text{termination of reaction}}\text{-}\text{A}_{\text{control group}}}\text{×}\text{ }\text{100\%}$ (2)

where A_experimental group_ represents the absorbance of the incubation medium in the experimental group at 540 nm after reacting with DNS reagent, A_control group_ represents the absorbance of the incubation medium in the control group at 540 nm after reacting with DNS reagent, A_termination of reaction_ represents the absorbance of the incubation medium in the experimental group at 540 nm after reacting with DNS reagent at the end of the reaction.

***Gravimetric method***: The biodegradation kinetics of the hydrogel were evaluated *via* a gravimetric protocol. Specifically, seven independent groups of hydrogel samples (1 mL each) were prepared. Each group (n=5) underwent an initial freeze-drying cycle to determine the baseline dry weight (*W*_0_). Subsequently, the freeze-dried hydrogels were individually incubated in PBS (37°C) under sterile conditions, with each group assigned to a distinct degradation timepoint (2, 4, 6, 8, 10, 12, or 14 days). After incubation, the hydrogels were retrieved, subjected to a second freeze-drying process, and reweighed to obtain the residual dry weight (*W*_t_). The degradation ratio for each timepoint was calculated using the Equation:

$\text{De}\text{g}\text{radation}\text{ ratio \%}\text{ }\text{=}\frac{{(W}_{t}{-W}_{0})}{W_{0}}\text{×}\text{ }\text{100 \%}$ (3)

**TTPy-NH_2_ release kinetics from SAH**

SAH was incubated in an equal volume (1 mL) of HAase solution (2 to 10 mg/mL) immediately after preparation. The incubation temperature was 37℃. The incubation solution was taken at intervals (0, 6, 12, 24, 48 and 72 h) to detect the concentration of TTPy-NH_2_ using the microplate reader (SynergyH1, biotech, USA) at 480 nm.

Furthermore, we investigated the correlation between swelling behavior and antimicrobial photosensitizer (TTPy-NH_2_) release in hydrogel wound dressings SAH. Hydrogel samples (1 mL) were immersed in three solutions: simulated body fluid (SBF, mimicking physiological ion composition), phosphate-buffered saline (PBS, representing isotonic conditions), and double-distilled water (ddH₂O, as a hypotonic control), to comprehensively assess performance across simulated biological environments. At predetermined intervals (0.5, 1, 2, 3, 4, 5, 6, 12 h), hydrogels were removed, surface-blotted with filter paper, and weighed to calculate swelling ratio (SR) using Equation (1) shown above. Concurrently, 200 μL aliquots of incubation media were sampled and analyzed *via* microplate reader at 470 nm (*λ*_max_ of TTPy-NH_2_) to quantify drug release. Swelling-drug release correlations were mathematically modeled using OriginPro 2023, with goodness-of-fit evaluated by coefficient of determination (R²), where R² =1 indicates strong functional dependence and R² =0 suggests independence.

**ROS detection of TTPy-NH_2_ loaded in SAH**

To evaluate the ROS generation capacity of TTPy-NH₂ loaded within the SAH hydrogel, a standardized DCFH-DA-based protocol was adapted for hydrogel matrices. Hydrogel samples containing 1 μM TTPy-NH₂ were prepared in a defined volume of 212 μL to ensure equivalent photosensitizer concentration to the aqueous solution control (detailed in *ROS detection of TTPy-NH₂ in aqueous solution*). Prior to testing, the pre-hydrolyzed DCFH solution (5 μM in PBS, pH 7.4) was uniformly infused into the hydrogel by immersion for 10 min under dark conditions. The ROS-sensitive hydrogel-DCFH composite was then irradiated with white light (16 mW/cm²), and time-dependent fluorescence emission at 525 nm (excitation: 485 nm) was monitored using a fluorescence spectrophotometer (FS5, Edinburgh Instruments) at 5-min intervals.

**Biocompatibilities of TTPy-NH_2_ and SAH**

The L929 cell line (mouse epithelioid fibroblasts cell line) was used for the *in vitro* studies. L929 cells were cultured in the MEM medium supplied with 10% horse serum and 1% penicillin−streptomycin with an incubator (37°C, 5% CO_2_). The cell culture medium was changed every other day, and cells were passaged upon 80% confluent. The cell activity was represented by the reduction rate of alamar blue (AB, S9022, warbio, China), which is a redox indicator related to cell proliferation. The reduction rate of alamar blue (RAAB) is calculated by the following Equation:

$\text{RAAB \%}\text{ }\text{=}\text{ }\frac{\text{E}_{\text{600}}\text{×}\text{OD}_{\text{570}}\text{-}\text{E}_{\text{570}}\text{×}\text{OD}_{\text{600}}}{\text{ }\text{E}_{\text{570}}\text{'×}\text{OD}_{\text{600}}\text{'-}\text{E}_{\text{600}}\text{'×}\text{OD}_{\text{570}}\text{'}}\text{×100\%}$ (4)

Where E_600_ = 117216 (extinction coefficient of oxidized AB at 600 nm wavelength); E_570_ = 80586 (extinction coefficient of oxidized AB at 570 nm wavelength); E_600_' = 14652 (extinction coefficient of reduced AB at 600 nm); E_570_' = 155677 (extinction coefficient of reduced AB at 570 nm); OD_600_ is the absorbance value of the sample measured at 600 nm. OD_570_ is the absorbance value of the sample measured at 570 nm. OD_600_' or OD_570_' is the absorbance of pure AB medium at 600 nm or 570 nm.

For validating the biocompatibility of TTPy-NH_2_, 5 × 10^4^ cells/well of L929 cells were seeded into a 24-well plate 1 day prior to the study. After 24 h, TTPy-NH_2_ was added to the cell with a final concentration of 0, 4, 6, 10, 16 µg/mL, in which 0 µg/mL was treated as the control group. The light group was set up to be illuminated with a lamp (16 mW/cm^2^) for 20 mins when TTPy-NH_2_ was added and the dark group was wrapped with foil. The hydrogels were sterilized via a modified pasteurization protocol comprising three consecutive thermal cycles (30 min at 60°C followed by 30 min at 4°C). For validating the biocompatibility of SAH, 5 × 10^4^ cells/well of L929 cells were seeded into a 24-well plate 1 day prior to the study. Three experimental groups were set in this study. Group 1 (HAase only): SAH was added to the well plate with different concentrations of HAase (2, 4, 6, 8 and10 µg/mL), and then the well plate was immediately sealed with foil to avoid light for 12, 24, 48 and 72 h. Group 2 (light only): SAH was added to the well plate, but no HAase was added. After incubation for 12, 24, 48 and 72 h, the cells were irradated with light (16 mW/cm^2^) for 20 mins. Group 3 (HAase + light): SAH was added to the well plate with different concentrations of HAase (2, 4, 6, 8 and10 µg/mL). After incubation for 12, 24, 48 and 72 h, the cells were irradated with light (16 mW/cm^2^) for 20 mins.

After the above treatments, the medium in the well plate was removed and replaced with alamar blue working solution (alamar blue reagent: MEM complete medium = 1: 10). After incubation for 4 h at 37°C, the absorbance of sample at 570 nm and 600 nm was read using a microplate reader (SynergyH1, biotek, USA), and the RAAB was calculated using Equation 4. The cell viability was calculated using the following Equation:

$\text{Cell viability \%}\text{ }\text{=}\text{ }\frac{\text{RAAB of experimental group }}{\text{RAAB of control group}}\text{×100\% }$ (5)

In addition, the morphologies of the cells were observed with a microscope in bright field.

**The hemocompatibility of SAH**

The hemocompatibility of the hydrogel was thoroughly evaluated under both light and dark conditions to comprehensively assess its biosafety in direct contact with blood. In the experimental design, three groups hydrogel with different volumes (50 μL, 100 μL, and 200 μL) were incubated in 12-well plates with 1 mL of 4% murine erythrocyte suspension. Each volume group was tested under two conditions: ​​light irradiation​​ (simulating therapeutic activation) and ​​darkness​​. Positive and negative controls were included using 500 μL ddH₂O and 500 μL PBS, respectively, mixed with an equivalent erythrocyte suspension. After 1 h of incubation at 37°C, hemoglobin release was quantified spectrophotometrically and the hemolysis ratio was calculated using the following equation:

$\text{Hemolysis }\text{ratio \%}\text{ }\text{=}\frac{{OD}_{\mathrm{sample}}{-OD}_{\mathrm{negative}}}{{OD}_{\mathrm{positive}}{-OD}_{\mathrm{negative}}}\text{×}\text{ }\text{100 \%}$ (6)

where the OD_sample_ is the absorbance of the sample group (540 nm), the OD_negative_ is the absorbance of the negative control group (540 nm), and the OD_positive_ is the absorbance of the positive control group (540 nm)^[6]^.

**Exploration of the regulation mechanism of Ca^2^ with HAase.**

**The impact of Ca^2+^ concentration on HAase activity**

A volume of 100 µL CaCl_2_ solution with different concentrations was added to 400 µL HA solution, followed by adding 500 µL HAase solution. The final concentrations of HA and HAase were 2 mg/mL and 2 mg/mL, respectively. The final concentrations of CaCl_2_ solution were 0, 5, 10, 25, 50, 60, 80, 100 mM. The above solution was placed in a 37℃ incubator for 30 mins. The concentration of reducing sugar produced by the degradation of HA was detected by 3,5-dinitrosalicylic acid (DNS) chromogenic method. Specifically, the samples above were mixed with DNS reagent (Solarbio) at a volume ratio of 1:2 and then boiled for 5 mins, cooled to room temperature and then read at 540 nm using a microplate reader (SynergyH1, biotech, USA). The activity of HAase can be calculated by the following Equation:

$\text{HAase activity \%}\text{ }\text{=}\frac{{\text{ }\text{A}}_{\text{experimental group}}}{\text{A}_{\text{control group}}}\text{×100\%}$ (7)

**Circular dichroism (CD) analysis**

CD measurements were performed to study the effect of Ca^2+^ on the secondary structure of HAase using a circular dichroism spectrometer (J815, Jasco, Japan). The preparation of the mixed solution of Ca^2+^ and HAase was the same as above. A quartz cuvette with a path length of 0.1 cm was utilized. The experiments were conducted at 20℃ with triplicates. The scanning speed was set at 200 nm/min. The scans of phosphate buffer solution used as a blank were recorded under the same conditions and subtracted from the experimental spectra. To interpret the secondary structure, the spectral data ranging from 180 to 240 nm were analyzed using the single spectrum analysis with a scale factor of 1 on the BeStSel web server software (ELTE Eötvös Loránd University, Budapest, Hungary)^[7]^.

**Molecular simulation studies**

Molecular simulations were performed to calculate the effect of Ca^2+^ on the three-dimensional conformation of HAase. Firstly, the nucleic acid sequence of HAase was obtained from the National Center for Biotechnology Information (NCBI, USA). Then, the nucleic acid sequence was translated into amino acid sequence using the MEGA6.0 software (The Pennsylvania State University, USA). The resulting amino acid sequence of HAase was constructed into tertiary structure using the Swissmodel software (Swiss Institute of Bioinformatics, Switzerland). The Ramachandran plot was performed by visualising the dihedral angles ψ and φ of the amino acid residues in the main chain of the protein structure to evaluate the rationality of the constructed protein tertiary structure. Next, the constructed tertiary structure of the HAase was used as the native structure for molecular docking, which was processed with the AutoDock Tools 1.5.6 software (The Scripps Research Institute, La Jolla, USA) to preserve the original charge of the protein. Then, the above structure along with the structure of Ca^2+^ as the ligand were used to calculate PM3 atomic charges for subsequent molecular docking using the algorithm Molecular Orbital PACkage (MOPAC, Stewart Computational Chemistry, USA)^[8]^. The prepared structures were exported as PDQBQT files for molecular docking, which was performed using the AutoDock 4.2.6 software package (The Scripps Research Institute, La Jolla, USA). The centre coordinates of the docking box were set to the centre of the protein and wrapped around the whole protein, the number of lattice points in each direction of XYZ was set to 50 Å × 50 Å × 50 Å, the number of docking times for each compound with the protein was set to 100, and the rest of the parameters were used as default values. The Pymol software (DeLano Scientiﬁc LLC, Waren DeLano, California) was used to plot the docking results derived from AutoDockTools. In addition, the hydrophilicity and hydrophobicity of HAase were analysed by the ProtScale (Swiss Institute of Bioinformatics, Switzerland) and Proteintools (Universität Bayreuth, Germany) softwares using the amino acid sequence of HAase.

**Three-dimensional fluorescence spectra**

The 3D fluorescence spectra were measured using a fluorescence spectrophotometer (F-4600, Hitachi, Japan) with a 10 mm quartz cuvette and a 150 W xenon lamp. The photomultiplier tube voltage, excitation/emission slit widths and scanning speed were set to 600 V, 10 nm and 1200 nm/min, respectively. Emission (Em) was set to 200-800 nm. Excitation (Ex) was set to 200-400 nm.

***In vitro* antibacterial properties**

**The amount of HAase produced by bacteria**

The amount of HAase that produced by different bacteria was explored. Firstly, a standard curve of HAase concentration versus reducing sugar concentration was studied. Specifically, HAase solutions at concentrations of 0.1, 1, 2, 4, 6, 8 and 10 mg/mL were added to HA solutions (1 mL) at a concentration of 2 mg/mL and with a volume ratio of 1:1. After incubation at 37°C for 2 h, the concentrations of reducing sugars produced by the HA degradation was measured using the DNS method Subsequently, the bacterial solution with concentrations of 10^2^, 10^4^, 10^6^, and 10^8^ CFU/mL was added to the HA solution (1 mL) with a concentration of 2 mg/mL with a volume ratio of 1:1 and incubated at 37 °C for 2 h. The concentrations of reducing sugar produced after HA was degraded by bacteria were detected by DNS method. The amount of HAase produced by bacteria at different concentrations was calculated from the standard curve.

**Antibacterial properties of TTPy-NH_2_**

The antibacterial behaviors of TTPy-NH_2_ and hydrogels were investigated with *Escherichia coli* ATCC® 25922™ (*E. coli*, as a gram-negative bacterium), *Staphylococcus aureus* ATCC® 25923™ (*S. aureus*, as a gram-positive bacterium) and methicillin-resistant *Staphylococcus aureus* ATCC® 43300™ (MRSA, as a drug-resistant bacterium). The bacteria were first cultured (37°C, 5% CO_2_) in agarose solid medium (Add 25 g of LB broth (Gibco) and 15 g agar (Gibco) to 1 L deionized water and adjust pH to 7.4, after being sterilized, it was poured into a petri dish and used after cooling and curing.) until single colonies formed. Then single colonies were picked and inoculated into LB liquid medium (25 g/L LB broth (sangon biotech, china) solution, pH 7.4) and cultured in a shaker (MQD-alcell, China) (37°C, 200 rpm). When the absorbance of the bacterial solution at 600 nm was 1, the concentration was 2×10^9^ CFU/mL. 2×10^5^ CFU bacteria were dispersed in 200 μL of PBS and incubated with certain concentration of TTPy-NH_2_ (0, 0.5, 1.0, 2.0, 3.0, 5.0 µg/mL). The absorbance of the bacterial solution at 600 nm was recorded immediately when the TTPy-NH_2_ was added (OD1), which was regarded as control. A light group and a dark group were set up. The light group was irradiated by a lamp (16 mW/cm^2^) for 20 mins and the dark group was wrapped with foil. The groups were then incubated in a shaker for 12 h (37°C, 200 rpm), after which the absorbance of the bacterial solution at 600 nm was measured (OD2). The bacterial proliferation was calculated from the following Equation:

$\text{Bacterial proliferation \%}\text{ }\text{=}\text{ }\frac{\text{OD2-OD1}}{\text{OD1}}\text{×100\%}$ (8)

**Binding ability of TTPy-NH_2_ to bacteria**

After harvesting bacteria by centrifugation, 1 × 10^8^ CFU/mL of *E. coli, S. aureus* and MRSA were added with 1 mL PBS containing 10 × 10^-6^ M TTPy-NH_2_. After dispersion with vortex, the bacteria were incubated with TTPy-NH_2_ at 37 °C at a shaking speed of 200 rpm for 15 min. To capture fluorescence images, 1 µL of stained bacteria solution was transferred to a piece of glass slide and then covered by a coverslip. The images were collected using a confocal laser scanning microscope (LSM900, Carl Zeiss, Germany). Capture conditions: AIE-PSs: *λ*ex = 488 nm and *λ*em = 600–700 nm.

**Antibacterial properties of SAH**

(I) *Inhibition zone*: bacterial solution of 50 µL with a concentration of 10^4^ CFU/mL was evenly coated with agarose solid medium using a coating rod. SAH of 400 µL was placed on the agarose solid medium and incubated in an incubator (37 ℃, 5% CO_2_) for 24 h. Because of the antibacterial effect of SAH, an area where bacteria cannot grow is formed around the hydrogel, *i.e.,* inhibition zone. The size of the inhibition zone area can represent the antibacterial properties of the hydrogel. The area of inhibition zone was measured using ImageJ software. A control group designated as the bacterial free-growth group was established in this experiment, wherein bacterial cultures were maintained under standard conditions without any experimental interventions to serve as a baseline for comparative analysis.

(II) *Plating method*: To explore whether the SAH can release AIE PSs upon different bacteria concentrations to achieve the antibacterial effect, 400 µL of different concentrations of bacterial solution were co-incubated with 400 µL of hydrogel in a shaker (37°C, 200 rpm) for 2, 4, 8, 16 h.and then irradiated with a lamp (16 mW/cm^2^) for 20 mins Afterwards, a certain concentration of bacterial solution was taken and diluted to coat the plates, and then incubated for 12 h before the number of colonies on the plates was counted.

**Animal experiments**

All the animal experiments involved in this work were approved by the Administrative Committee on Animal Research in Shenzhen TOP Biotechnology Co., Ltd. (SYXK(YUE)2022-0293). Male BALB/c mice (18- 22 g) about 6-7 weeks old were obtained from animal laboratory center of Guangdong province and housed in SPF laboratory animal room. All the mice had completed quarantine before the experiment. After mice were anesthetized by isoflurane gas, the hair on their backs was shaved to expose the skin, followed by creation of full-thickness skin wound (5 mm) under sterile conditions. Then each defined skin wound of mice was inoculated with 10^4^ CFUs of *S. aureus*.

**Wound healing**

The above mice were randomly divided into different groups with n = 7. Groups were set as follows: Wound only, 3M tape (wounds were covered only with commercial tapes), TTPy-NH_2_ (TTPy-NH_2_ only, and the amount of TTPy-NH_2_ was equal with that in SAH), SAH w/o TTPy-NH_2_ (hydrogel only), SAH (TTPy-NH_2_-loaded hydrogel). All those 5 groups were provided with light illumination (16 mW/cm^2^).

The day of setting model and processing treatment was defined as the day 0. The wound area was recorded daily for the next 14 days. The wound healing rate was calculated according to the following Equation:

$\text{Healing rate \%}\text{ }\text{=}\text{ }\frac{\text{S0-St}}{\text{S0}}\text{ ×100\% }$ (9)

Where S0 is the size of the wound on day 0 and St is the size of the wound on the day of measurement. The size of the wound is identified and calculated using ImageJ software.

***In vivo* antibacterial effect**

From day 1, the wound sites were irradiated daily for 20 mins using a lamp (16 mW/cm^2^). The tissue fluid from the wound sites was collected by swabs and incubated in LB liquid medium for 12 h. The incubated bacterial fluid was subsequently diluted to a certain concentration for plate coating. Colonies were counted after a further 12 h of culture.

**Histopathological study**

On day 3, 7 and 14, the skin of the wound sites (including the entire wound adjacent to the normal skin) were collected (1.5 cm × 1.5 cm). All samples were fixed overnight in 4% paraformaldehyde, followed by paraffin embedding and ultrathin sectioning. Sections were subjected to hematoxylin-eosin (H&E) staining to understand the distribution and repair of infected wounds. Masson's trichrome staining was used to detect the collagen formation during the healing process. For immunohistochemistry, sections were deparaffinized and rehydrated, hydrogen peroxide (3%) was added to eliminate endogenous peroxidase, and bovine serum albumin (5%) was used to block nonspecific reactions. Then, the sections were incubated overnight with rabbit anti-CD31 (GB11063-2, 1: 500, Servicebio) antibodies, mouse anti-Ki67 (GB121141, 1: 500, Servicebio) antibodies, mouse anti-α-SMA (GB12045, 1: 3000, Servicebio) antibodies, rabbit anti-IL-10 (GB11534, 1: 500, Servicebio) antibodies and rabbit anti- TNF-α (GB11188, 1: 500, Servicebio) antibodies, respectively. This was followed by treatment with HRP goat anti-rabbit (GB23204, 1: 200, Servicebio) for 50 mins and visualization with a DAB kit (ZSGB-BIO, China). All slices were photo-captured by microscope (DMi8, Leica, Germany).

**Statistics.**

Quantitative data were expressed as mean ± standard deviation (SD). Significant differences were examined using one-way ANOVA.


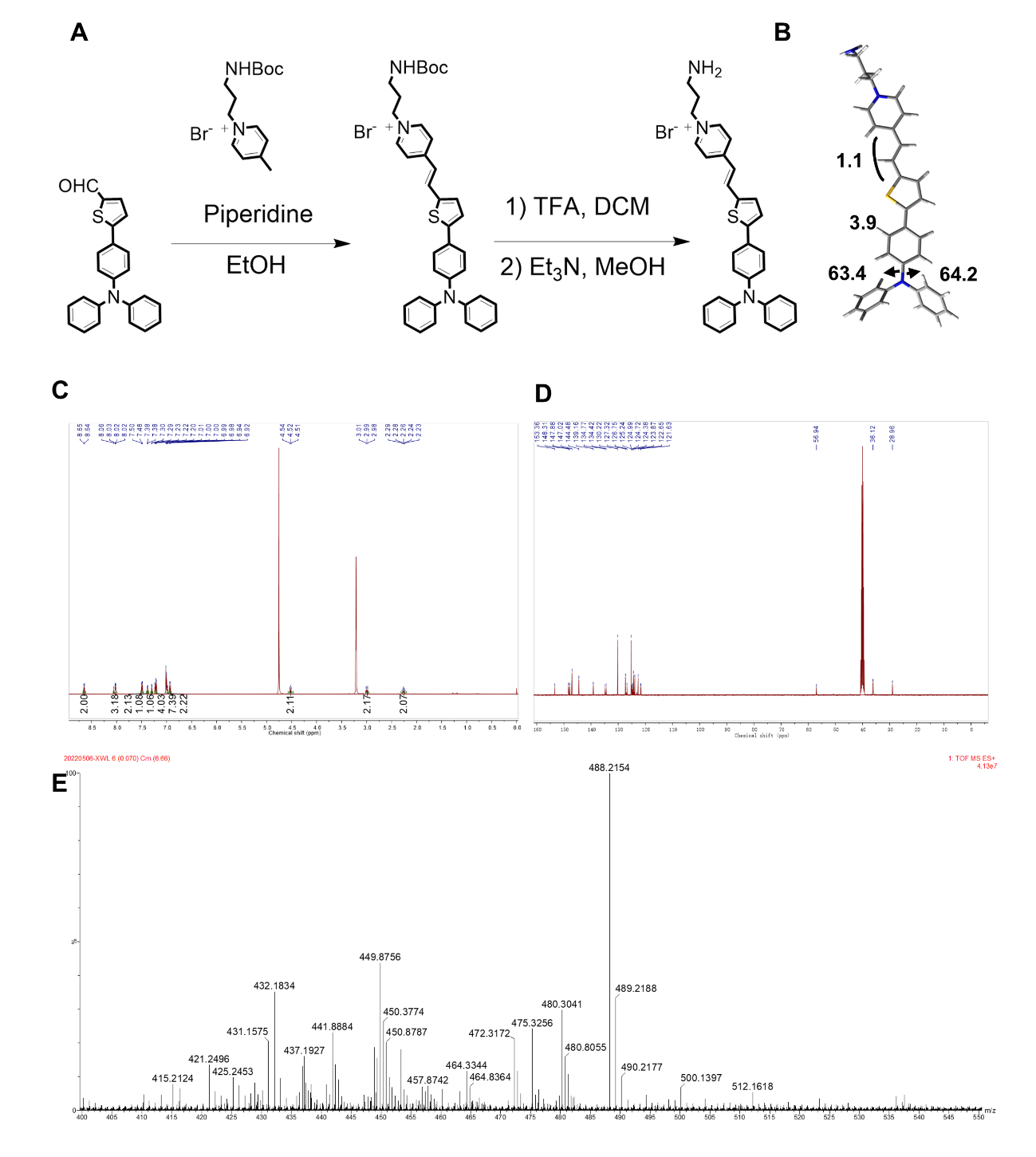


Figure S1. Synthesis and characterization of TTPy-NH_2_. **A**) Synthetic route of TTPy-NH_2_. **B**) Chemical structure and optimized ground state (S_0_) geometry. **C**) ^1^H NMR spectrum of TTPy-NH_2_ in MeOD. **D**) ^13^C NMR spectrum of TTPy-NH_2_ in DMSO-d_6_. **E**) ESI HRMS spectrum of TTPy-NH_2_.

**Supplementary discussion 1**

The structure and purity of TTPy-NH_2_ were verified by ^1^H NMR (Figure S1c), ^13^C NMR (Figure S1d) and HRMS (Figure S1e) with satisfactory results. ^1^H NMR (500 MHz, MeOD) δ 8.65 (d, *J* = 6.9 Hz, 2H), 8.03 (dd, *J* = 11.2, 8.9 Hz, 3H), 7.49 (d, *J* = 8.8 Hz, 2H), 7.38 (d, *J* = 3.9 Hz, 1H), 7.30 (d, *J* = 3.9 Hz, 1H), 7.25-7.17 (m, 4H), 7.04-6.96 (m, 7H), 6.93 (d, *J* = 8.8 Hz, 2H), 4.52 (t, *J* = 7.6 Hz, 2H), 3.07 (s, 1H), 3.05-2.92 (m, 2H), 3.01-2.95 (m, 2H), 2.33-2.18 (m, 2H). ^13^C NMR (100 MHz, DMSO-d_6_) δ 153.36 (s), 148.31 (s), 147.88 (s), 147.02 (s), 144.48 (s), 139.16 (s), 134.77 (s), 134.42 (s), 130.22 (s), 127.32 (s), 126.75 (s), 125.24 (s), 124.99 (s), 124.72 (s), 124.38 (s), 123.87 (s), 122.65 (s), 121.63 (s), 56.94 (s), 36.12 (s), 28.96 (s). ESI HRMS: calcd. for C_32_H_30_N_3_S [M-Br]^+^ : 488.2155, found: 488.2154.


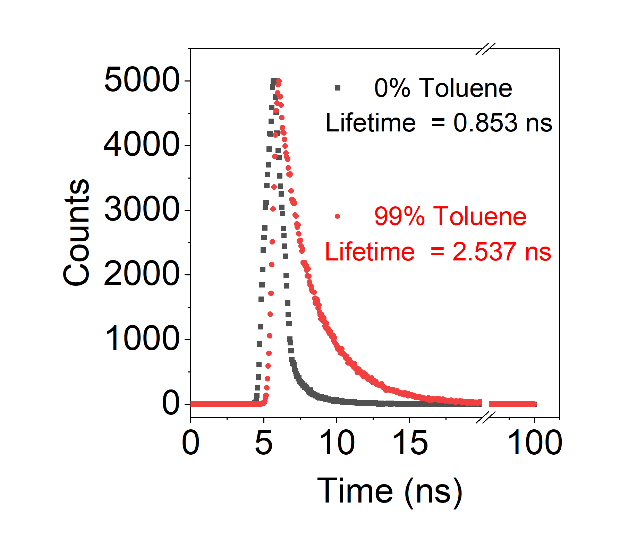


Figure S2. Time-resolved photoluminescence (TRPL) tests TTPy-NH_2_ in solvent with 0% and 99% of toluene.

**
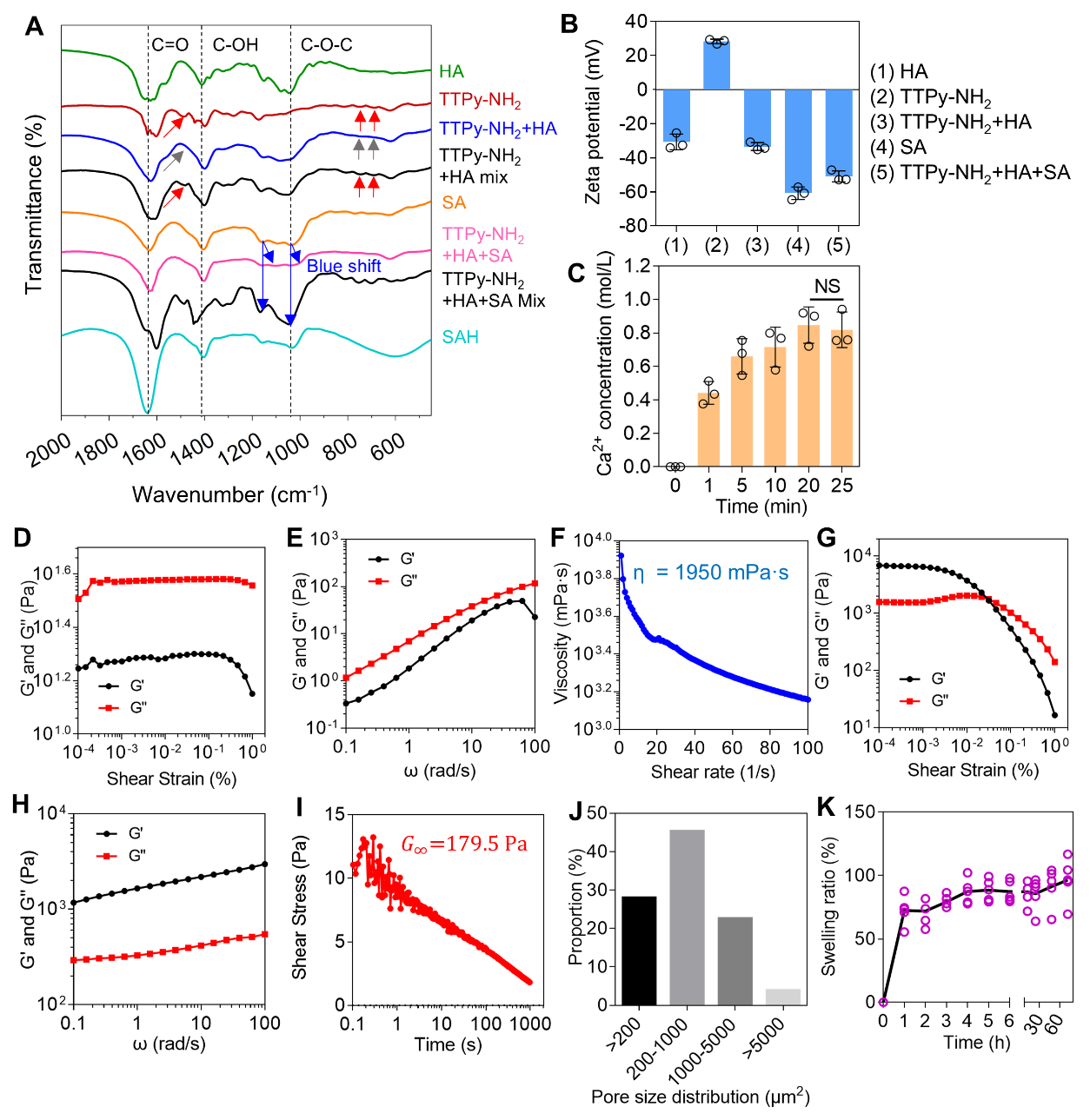
**

Figure S3. Characterization of SAH. **A**) FT-IR of HA, TTPy-NH_2_, TTPy-NH_2_ loaded in HA, TTPy-NH_2_ + HA mix (simple physical mixing of two powders in equal proportions), SA, precursor, TTPy-NH_2_ + HA + SA mix (simple physical mixing of three powders in equal proportions) and SAH. **B**) Zeta potential of HA, TTPy-NH_2_, TTPy-NH_2_ loaded in HA, SA, and the precursor (TTPy-NH_2_ + HA + SA) Effect of Ca^2+^ concentration on HAase activity. **C**) The concentration of Ca^2+^ in SAH during the preparation of SAH under different incubation time. **D**) Amplitude sweep, **E**) frequency sweep, and **F**) viscosity curve of the hydrogel precursor solution. **G**) Amplitude sweep, **H**) frequency sweep and **I**) stress relaxation test of the SAH hydrogel wound dressing. **J**) Analysis of the microstructural pore size distribution of the SAH. **K**) The swelling ratio of SAH incubated in PBS for different time.


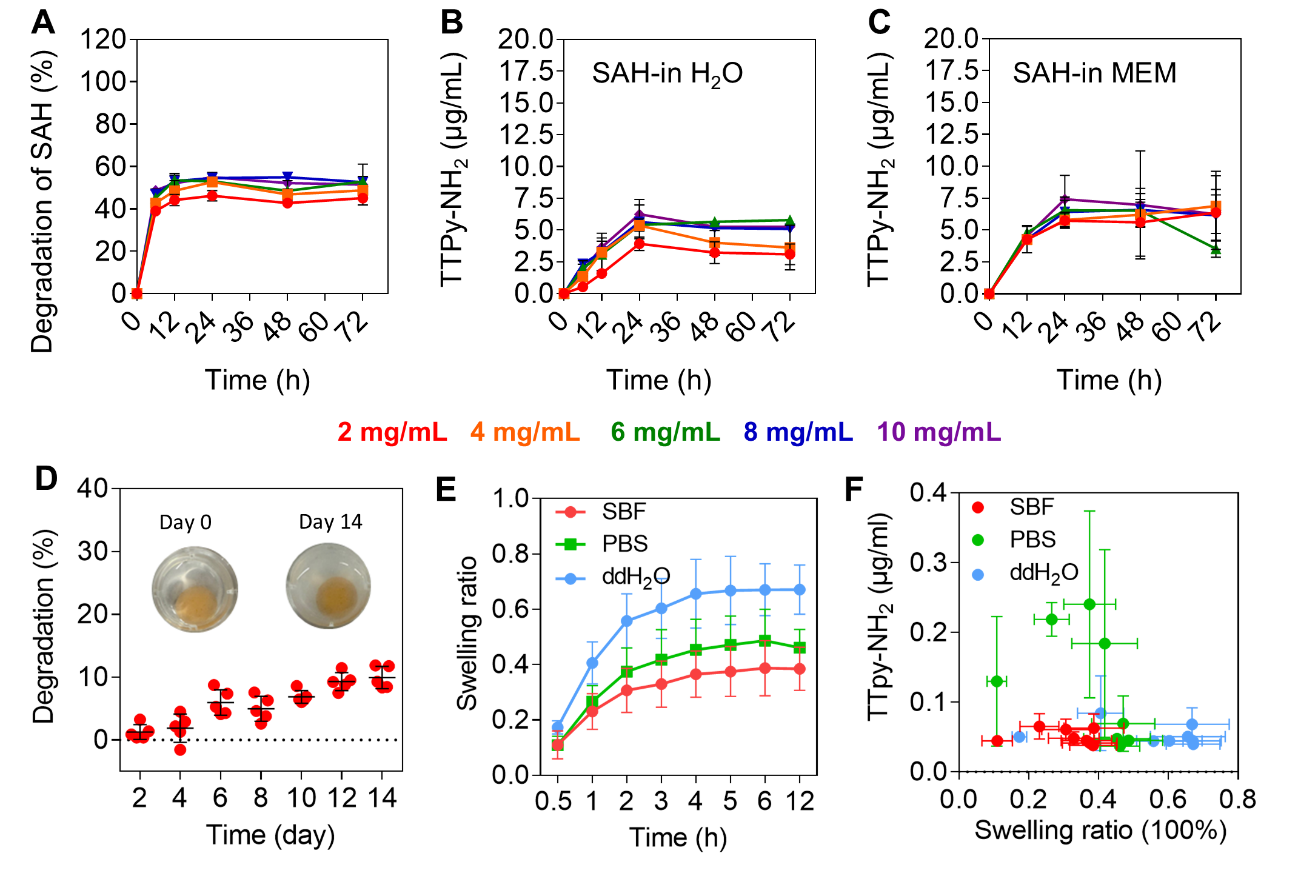


Figure S4. The degradation and TTPy-NH_2_ release kinetics at different HAase concentration of SAH. **A**) Degradation of SAH with HAase (2-10 mg/mL). **B**) Release of TTPy-NH_2_ from SAH with HAase (2 to 10 mg/mL) in water at 37 °C. **C**) Release of TTPy-NH_2_ from SAH with HAase (2-10 mg/mL) in MEM complete medium at 37 °C. **D**) The degradation rate of the hydrogel wound dressing SAH in PBS using the gravimetric method. **E**) Relationship between swelling ratio and the released TTPy-NH_2_ from SAH. **F**) Functional correlation between swelling ratio and the released TTPy-NH₂ amount.


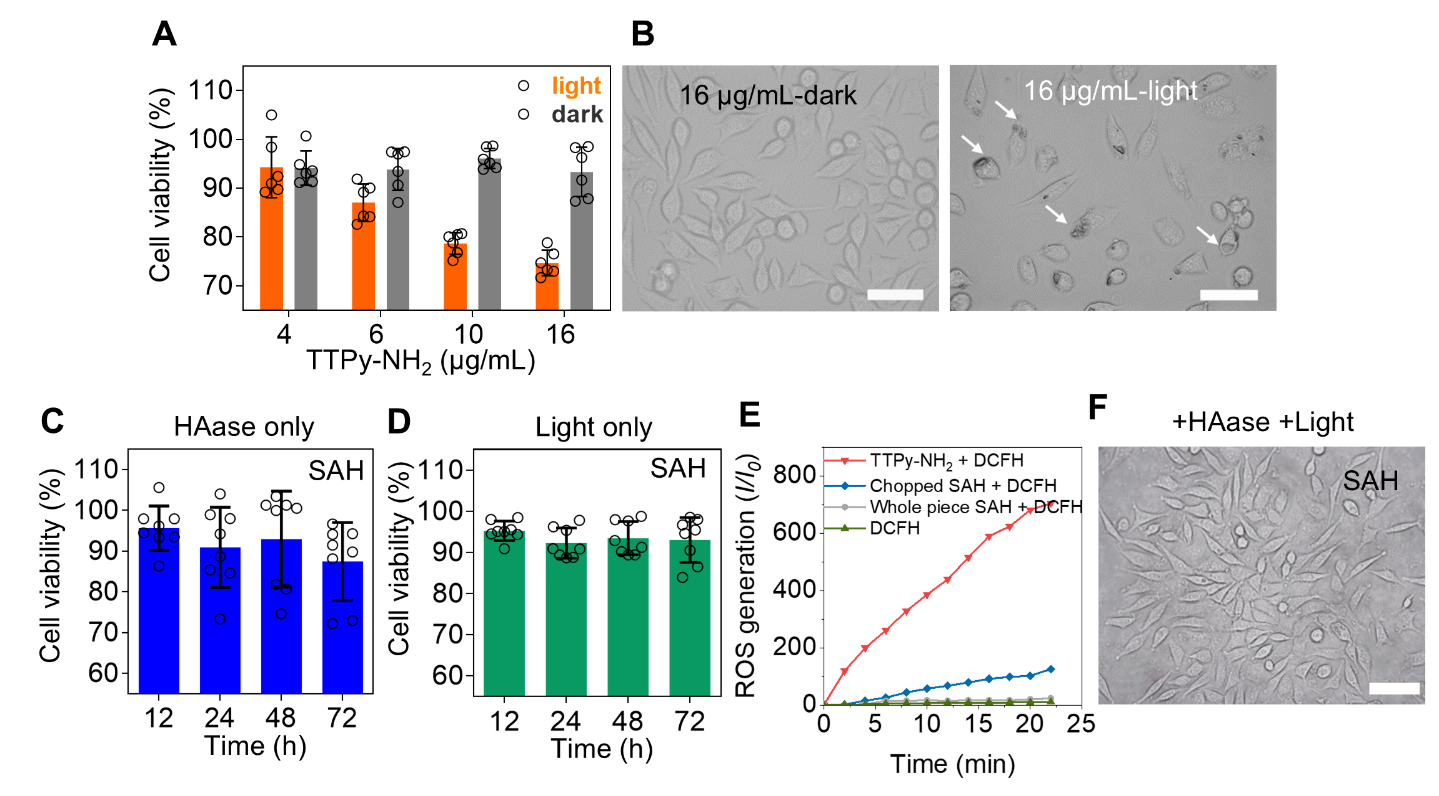


Figure S5. Biocompatibilities of TTPy-NH_2_ and SAH. **A**) The cell viability of L929 co-cultured with different concentrations of TTPy-NH_2_ under both light and dark conditions. **B**) Morphology of the cells treated with 16 mg/mL of TTPy-NH_2_ under both light and dark conditions. The white arrows indicate the abnormal intracellular bubbles, scale bar: 30 μm. The cell viability of L929 co-cultured with SAH under different culture conditions: **C**) HAase only (10 mg/mL), **D**) light only (16 mW/cm^2^), The cell viability of L929 cells tested by Alamar blue assay (n = 9). **E**) ROS generation efficiency of TTPy-NH_2_ (1 μM) at different conditions upon visible light irradiation indicated by relative changes in PL intensity of DCFH. **F**) Morphology of the cells treated with HAase (10 mg/mL) and light irradiation (16 mW/cm^2^). Scale bar: 50 μm.


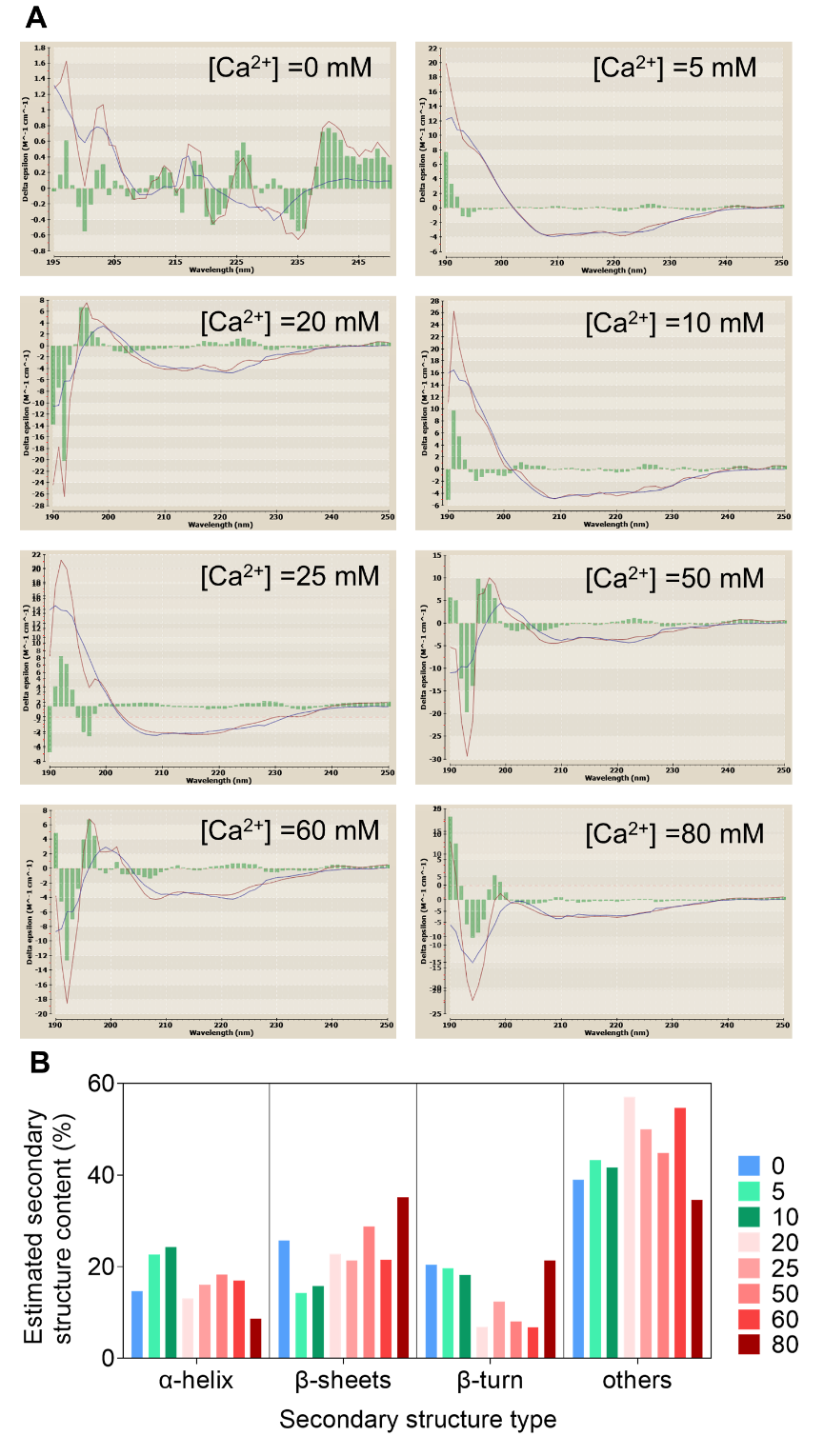


Figure S6. The single circular dichroism spectra analysis of Ca^2+^-treated HAase with a scale factor of 1 on the BeStSel software. **A**) Results of fitting (blue line) and calculation (green column) of experimental CD spectral data (red line). **B**) The estimated secondary structure content (%) of HAase treated with different concentration of Ca^2+^ (0-80 mM).


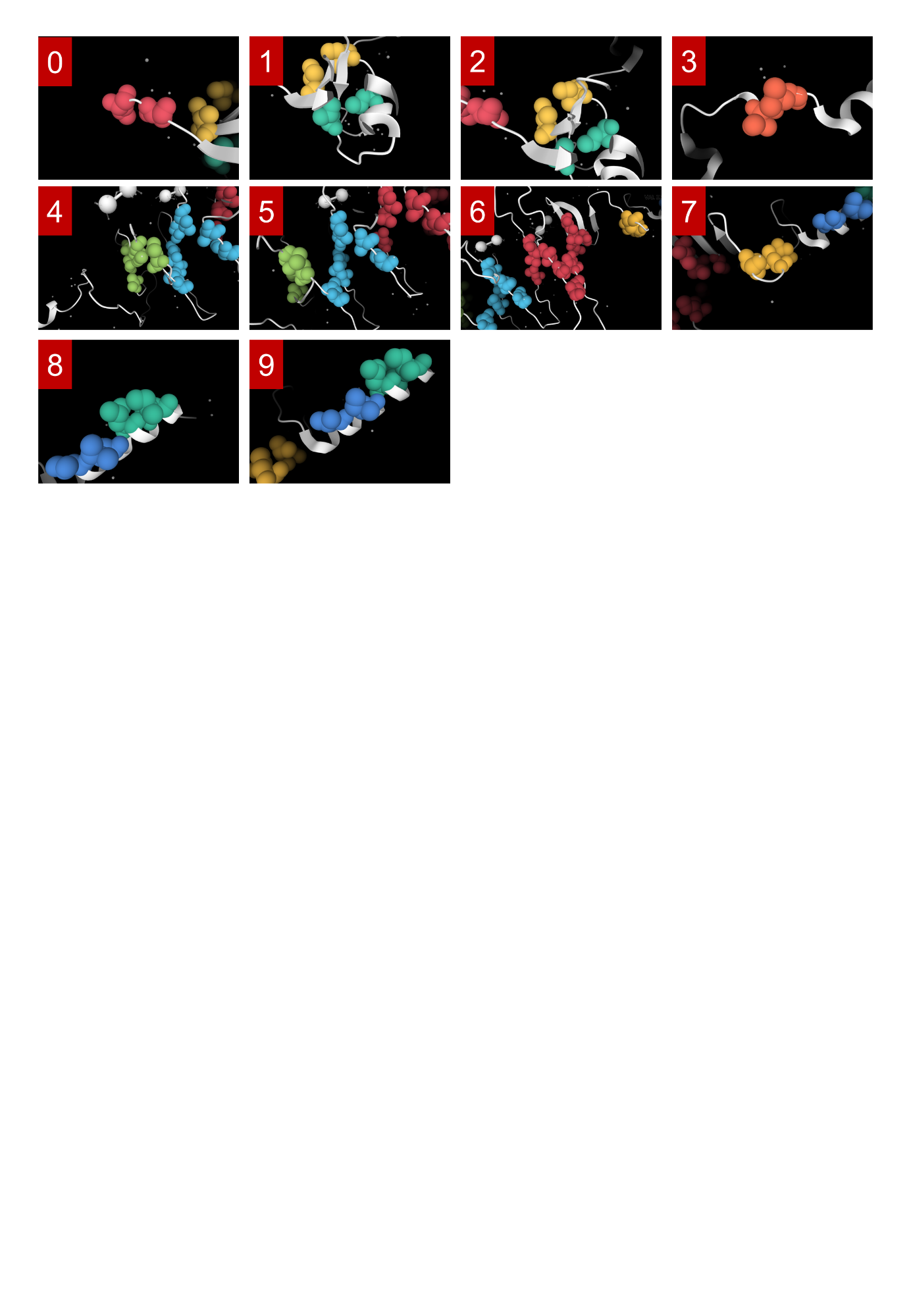


Figure S7. Ten specific hydrophobic clusters in the tertiary structure of HAase obtained by the Proteintools software.


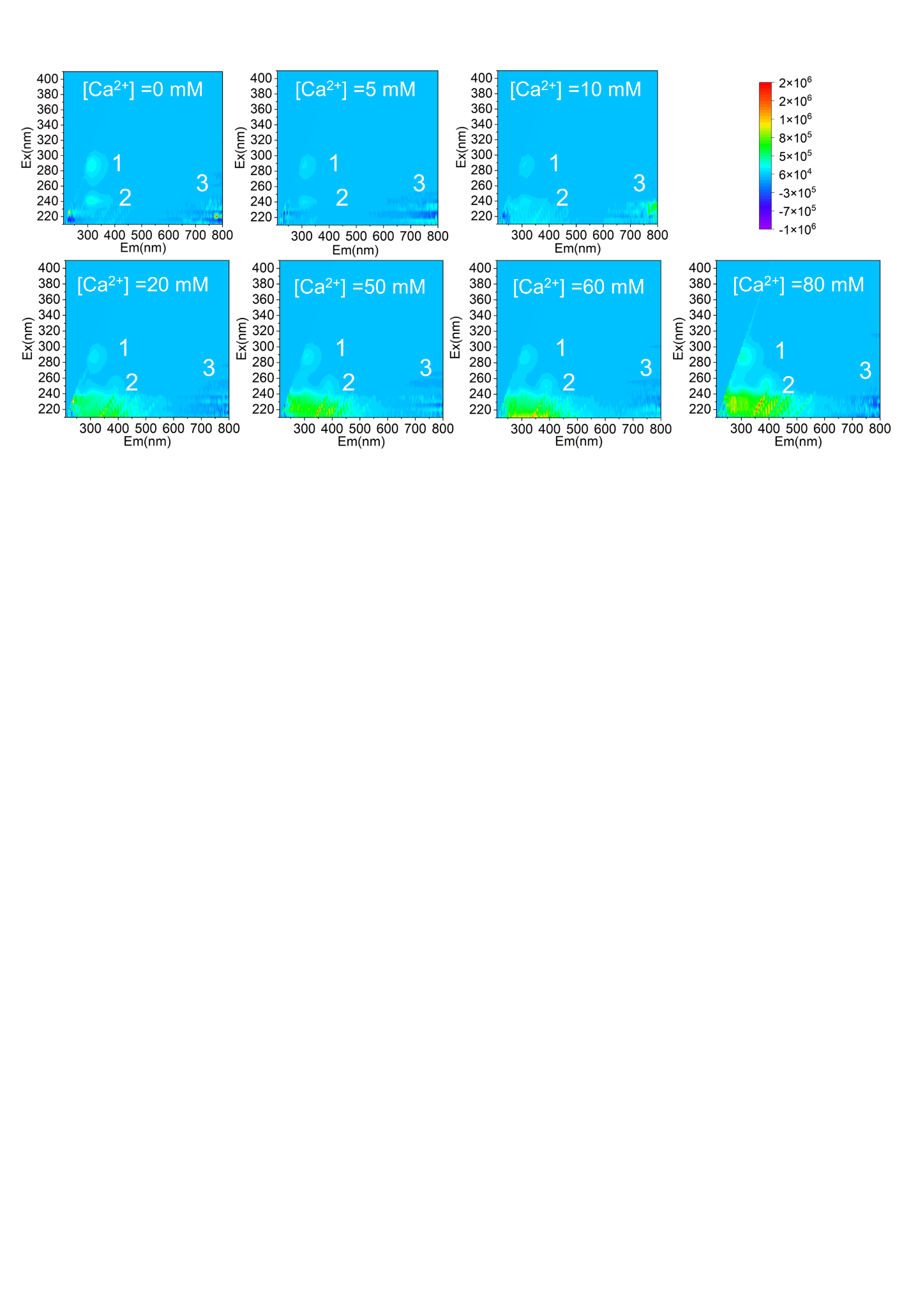


Figure S8. The three-dimensional ﬂuorescence maps of HAase with different concentrations of Ca^2+^ (0-80 mM), T = 298K, the rayleigh scattering peaks (Ex = Em) have been artificially removed.


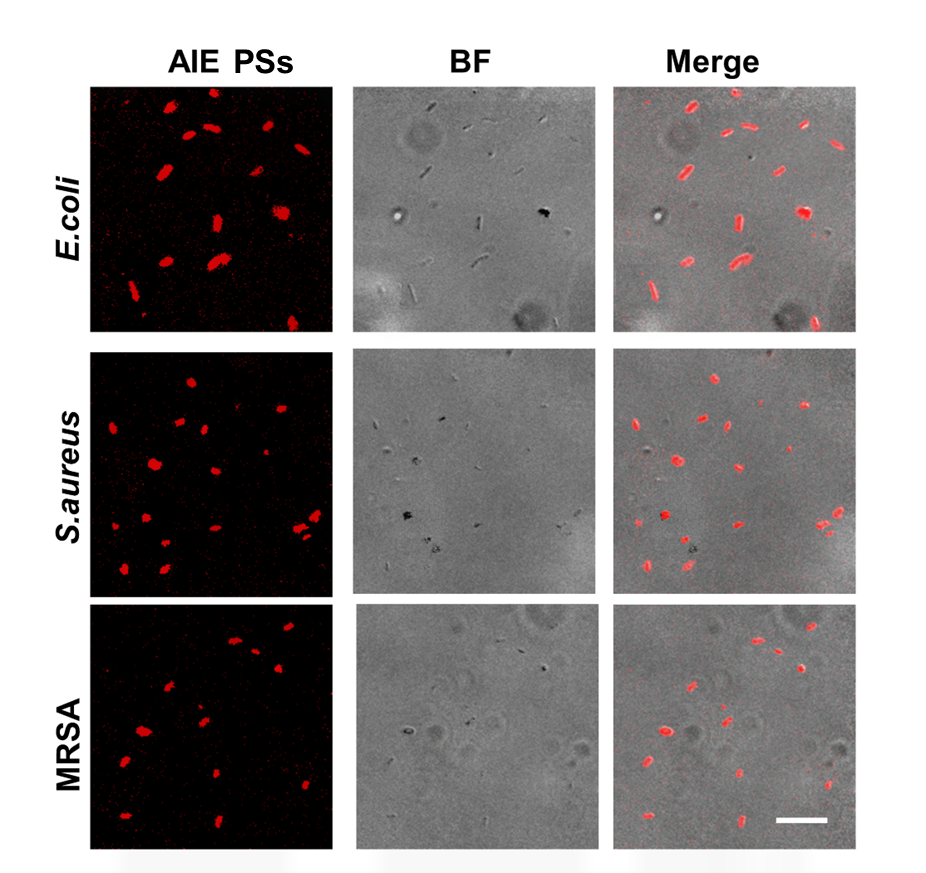


Figure S9. Confocal laser scanning microscopy (CLSM) images of bacteria after treated with AIE PSs (TTPy-NH_2_, 10×10^-6^ M). BF: bright field. Ex = 488 nm, Em = 600-700 nm. Scale bar: 5 μm.


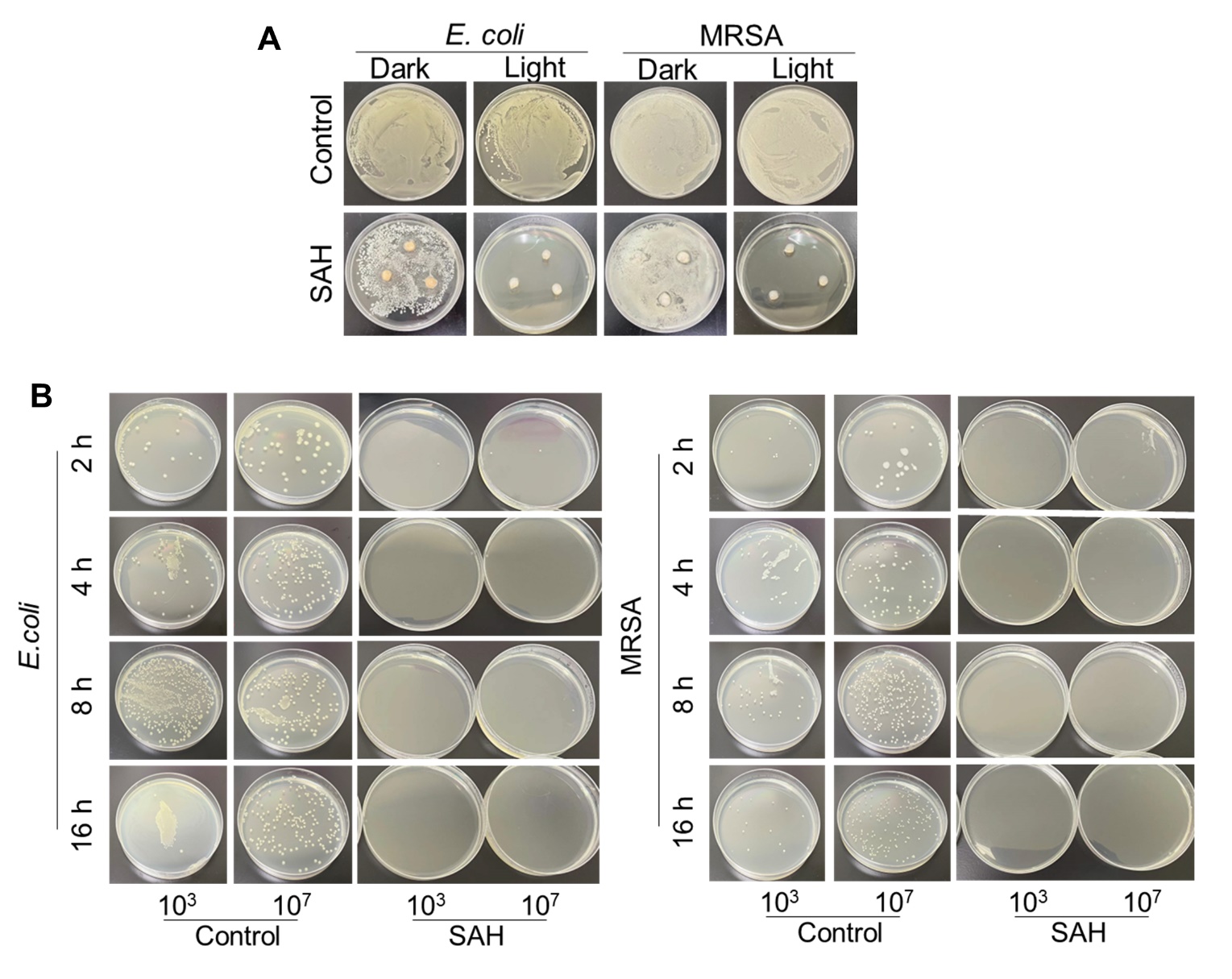


Figure S10. *In vitro* antibacterial abilities of SAH. **A**) The antibacterial properties of SAH characterized by the inhibition zone assay. **B**) The antibacterial properties of SAH against *E. coli* and MRSA at high (10^7^ CFU/mL) and low (10^3^ CFU/mL) concentrations.


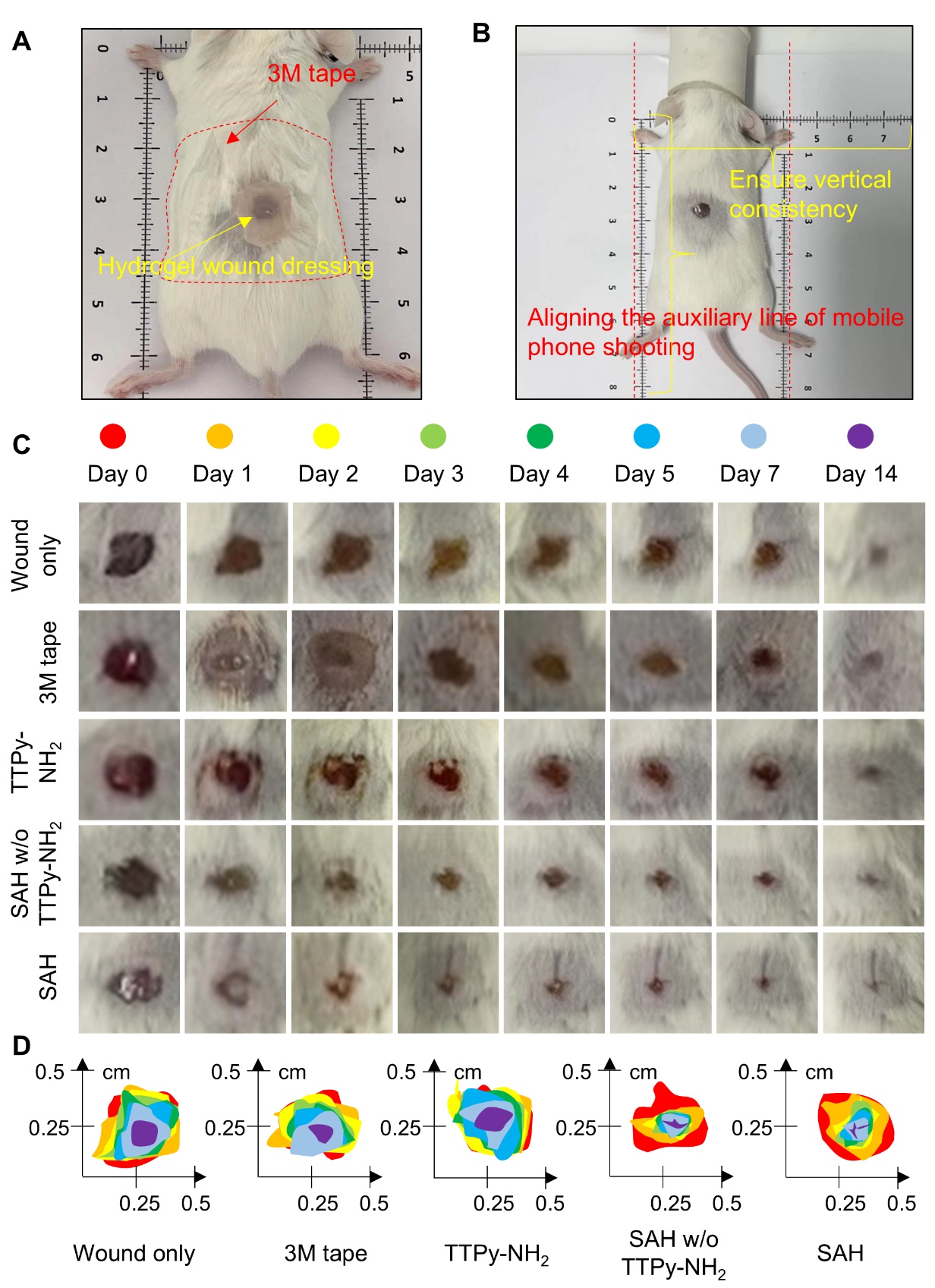


Figure S11. *In vivo* healing effect of the infected wounds. **A**) Illustration of the method for fixing hydrogel wound dressings on the wound site of mice in animal experiments. **B**) The method employed in this study to ensure consistency in wound photography records of mice during animal experiments. **C**) Representative pictures of wound sites in different groups on day 0-5, 7 and 14. **D**) Traces of wound-bed closure on day 0-5, 7 and 14.


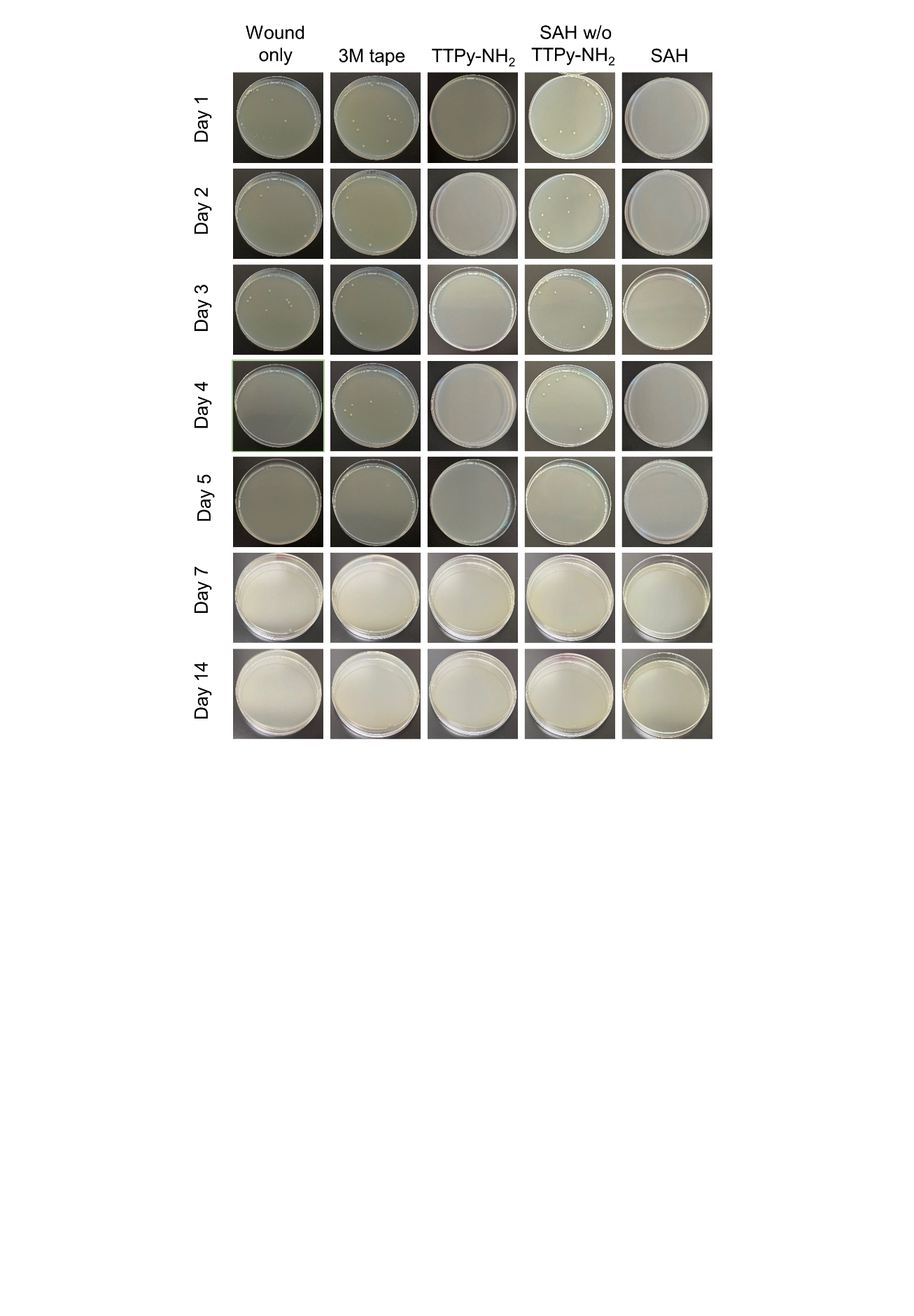


Figure S12. The plate culture images of bacteria sampled from the tissue fluid at wound sites. Bacteria were taken from the tissue fluid in wound sites using a cotton swab and cultured on day 1-5, 7, 14.


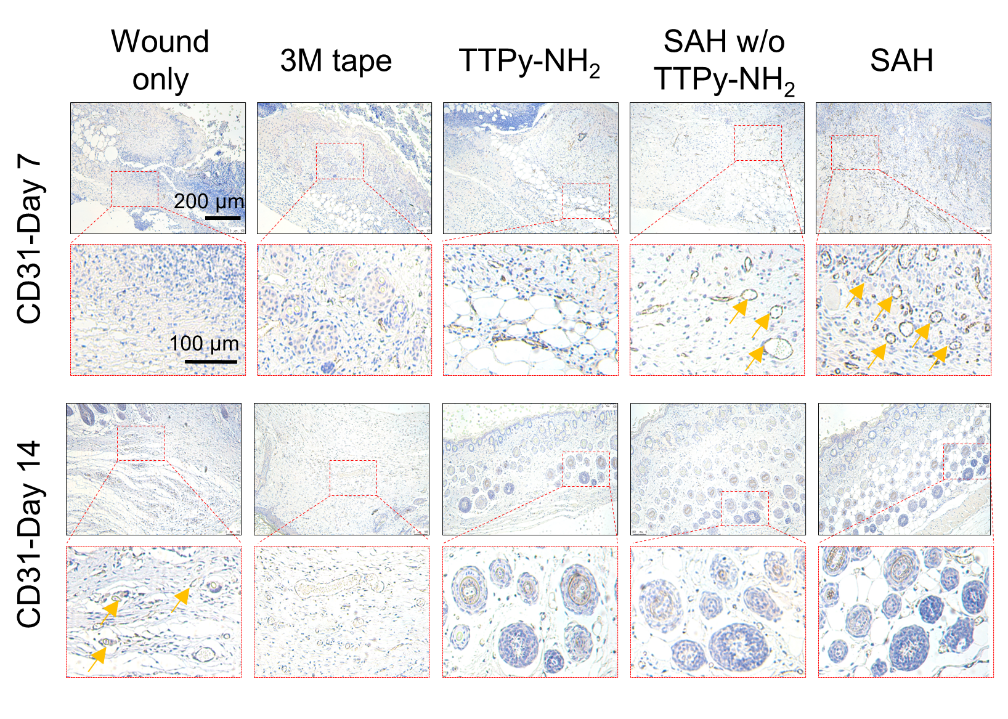


Figure S13. The expression levels of CD31 in different groups characterized by immunohistochemical staining. Yellow arrows indicate the early neovascularization.

Table S2. Results of molecular docking and scoring of HAase with Ca^2+^.

| **Position** | **Binding Energy (kcal/mol)** |
| --- | --- |
| **1** | -1.287 |
| **2** | -1.082 |
| **3** | -0.971 |
| **4** | -0.961 |
| **5** | -0.960 |
| **6** | -0.958 |

Table S3. Information of hydrophobic clusters of HAase.

| **Cluster ID** | **Area** | **Number of contacts** | **Contacts/Residue** | **Area/Residue** |
| --- | --- | --- | --- | --- |
| 0 | 96.19 | 2 | 1 | 48.09 |
| 1 | 51.42 | 2 | 1 | 25.71 |
| 2 | 96.19 | 2 | 1 | 48.09 |
| 3 | 96.41 | 2 | 1 | 48.20 |
| 4 | 484.26 | 10 | 1.67 | 48.43 |
| 5 | 630.31 | 12 | 1.71 | 52.53 |
| 6 | 1265.50 | 26 | 2.17 | 48.67 |
| 7 | 307.40 | 4 | 1.33 | 76.85 |
| 8 | 210.10 | 5 | 1.67 | 42.02 |
| 9 | 41.89 | 2 | 1 | 20.94 |

**References**

[1] Y. Wang, D. Yan, L. Wang, D. Wang, B. Z. Tang, *Adv. Sci.* **2021**, 8, e2100811.

[2] U. D’Amora, A. Ronca, M. G. Raucci, S. M. Dozio, H. Lin, Y. Fan, X. Zhang, L. Ambrosio, *Regen. Biomater.* **2019**, 6, 249.

[3] Y. Chen, Y. Chen, X. Xiong, R. Cui, G. Zhang, C. Wang, D. Xiao, S. Qu, J. Weng, *Mater. Today Bio.* **2022**, 14, 100261.

[4] Z. Wei, M. Lei, Y. Wang, Y. Xie, X. Xie, D. Lan, Y. Jia, J. Liu, Y. Ma, B. Cheng, S. Gerecht, F. Xu, *Nat. Commun.* **2023**, 14, 8307.

[5] X. Zhang, Y. Li, Z. Ma, D. He, H. Li, *Bioact. Mater.* **2021**, 6, 3692.

[6] X. Meng, Y. Lu, Y. Gao, S. Cheng, F. Tian, Y. Xiao, F. Li, *Int. J. Macromol.* **2021**, 182, 512.

[7] X. Li, H. Liu, Z. Yang, H. Duan, Z. Wang, Z. Cheng, Z. Song, X. Wu, *J. Mol. Struct.* **2021**, 1241, 130686.

[8] J. J. P. Stewart, *J. Mol. Model.* **2017**, 23, 154.
